# Regulator of G protein signaling protein 6 alleviates acute lung injury by inhibiting inflammation and promoting cell self-renewal in mice

**DOI:** 10.1186/s11658-023-00488-z

**Published:** 2023-12-08

**Authors:** Juan Song, Miao Li, Cuicui Chen, Jian Zhou, Linlin Wang, Yu Yan, Jun She, Lin Tong, Yuanlin Song

**Affiliations:** 1 Shanghai Institute of Infectious Disease and Biosecurity, Fudan University, Shanghai, 200032 China; 2 Shanghai Key Laboratory of Lung Inflammation and Injury, Department of Pulmonary Medicine, Zhongshan Hospital, Fudan University, Shanghai, 200032 China; 3 Department of Pulmonary Medicine, Zhongshan Hospital (Xiamen), Fudan University, Xiamen, Fujian 361000 China; 4 Shanghai Respiratory Research Institute, Shanghai, 200032 China

**Keywords:** Acute lung injury, Regulator of G protein signaling 6, Inflammation, Cell-renewal, Apoptosis

## Abstract

**Background:**

Acute respiratory distress syndrome (ARDS) is a disease with high mortality and morbidity. Regulator of G protein signaling protein 6 (RGS6), identified as a tumor suppressor gene, has received increasing attention owing to its close relationship with oxidative stress and inflammation. However, the association between ARDS and RGS6 has not been reported.

**Methods:**

Congruously regulated G protein-coupled receptor (GPCR)-related genes and differentially expressed genes (DEGs) in an acute lung injury (ALI) model were identified, and functional enrichment analysis was conducted. In an in vivo study, the effects of RGS6 knockout were studied in a mouse model of ALI induced by lipopolysaccharide (LPS). HE staining, ELISA, and immunohistochemistry were used to evaluate pathological changes and the degree of inflammation. In vitro, qRT‒PCR, immunofluorescence staining, and western blotting were used to determine the dynamic changes in RGS6 expression in cells. The RGS6 overexpression plasmid was constructed for transfection. qRT‒PCR was used to assess proinflammatory factors transcription. Western blotting and flow cytometry were used to evaluate apoptosis and reactive oxygen species (ROS) production. Organoid culture was used to assess the stemness and self-renewal capacity of alveolar epithelial type II cells (AEC2s).

**Results:**

A total of 110 congruously regulated genes (61 congruously upregulated and 49 congruously downregulated genes) were identified among GPCR-related genes and DEGs in the ALI model. RGS6 was downregulated in vivo and in vitro in the ALI model. RGS6 was expressed in the cytoplasm and accumulated in the nucleus after LPS stimulation. Compared with the control group, we found higher mortality, more pronounced body weight changes, more serious pulmonary edema and pathological damage, and more neutrophil infiltration in the RGS6 knockout group upon LPS stimulation in vivo. Moreover, AEC2s loss was significantly increased upon RGS6 knockout. Organoid culture assays showed slower alveolar organoid formation, fewer alveolar organoids, and impaired development of new structures after passaging upon RGS6 knockout. In addition, RGS6 overexpression decreased ROS production as well as proinflammatory factor transcription in macrophages and decreased apoptosis in epithelial cells.

**Conclusions:**

RGS6 plays a protective role in ALI not only in early inflammatory responses but also in endogenous lung stem cell regeneration.

**Graphical Abstract:**

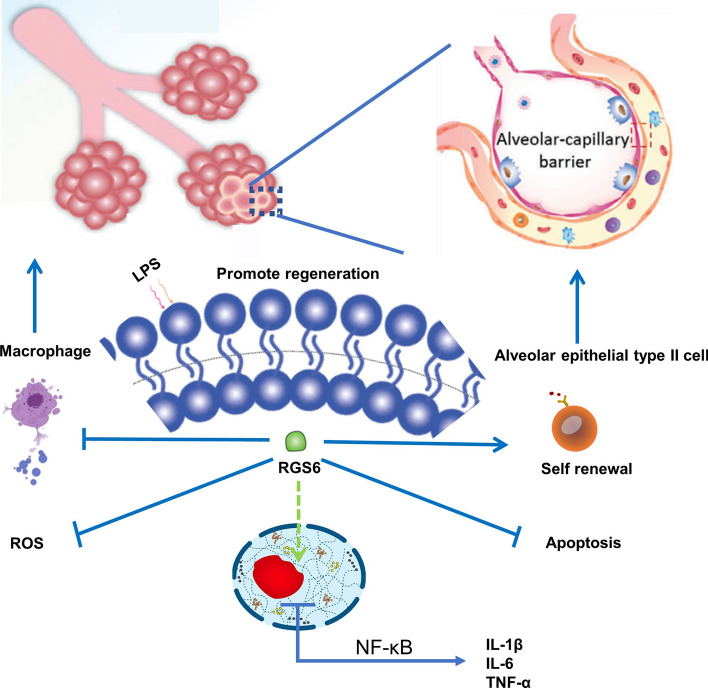

**Supplementary Information:**

The online version contains supplementary material available at 10.1186/s11658-023-00488-z.

## Background

Acute respiratory distress syndrome (ARDS) is a disease with high mortality and morbidity accompanied by some common clinical characteristics, such as dyspnea, cyanosis, and refractory hypoxemia, diagnosed on the exclusion of heart failure. In most patients, bilateral pulmonary infiltrates are visible on chest radiography or CT scans. Severe systemic infection or inflammation, inappropriate mechanical ventilation, severe pneumonia, aspiration, high-risk surgeries, and many other factors could contribute to ARDS. ARDS is a heterogeneous disease and exhibits different phenotypes among patients. Generally, uncontrolled inflammatory responses and impaired endogenous lung stem cell regeneration are considered the core pathophysiological mechanisms. To date, there are no efficient medications or treatments for ARDS other than symptomatic treatment and mechanical ventilation. Recently, some clinical studies revealed that ARDS patients could benefit from traditional Chinese medicine, aspirin, early treatment with inhaled budesonide and formoterol, neuromuscular blocking agents, hydrocortisone, etc. [1–5]. However, the effects of these treatments are limited. New therapeutic targets for ALI/ARDS, such as ferroptosis, extracellular vesicles, and pyroptosis, are constantly being discovered [6–8]. Therefore, it is worthwhile to explore and identify new therapeutic targets to improve the cure rate of ARDS.

Regulator of G protein signaling (RGS) proteins determine the magnitude and duration of G protein-coupled receptor (GPCR) signaling, which contributes to nearly every physiological process and many common diseases. GPCRs are a large group of membrane protein receptors and contain seven transmembrane α-helices in their stereo-structures as well as G protein binding sites. Once activated by extracellular agonists, conformational changes occur in GPCRs where the guanosine diphosphate (GDP) originally bound to the G protein is replaced by guanosine triphosphate (GTP), after which the G_α_ subunit in the G protein separates from the G_β_ and G_γ_ subunits. These processes activate the G protein (specifically the G_α_ subunit) to participate in the next step of signal transduction. GPCRs are important targets for drug design. RGS proteins serve as gatekeepers of signaling mediated by G proteins. Based on the protein domain structure, the 20 canonical RGS proteins are divided into four subfamilies. RGS proteins are considered multifunctional signaling regulators and play a wide range of roles in maintaining human health. For instance, RGS2 is involved in thyroid-stimulating hormone receptor (TSHR)-induced Gq signal transduction and inhibits TSHR signaling [9]. Mice lacking RGS10 and RGS18 showed reduced platelet survival and a 40% decrease in platelet count, which in turn modulated their hemostatic response to injury [10]. RGS1 deactivates G protein signaling and reduces macrophage chemotaxis in atherosclerosis and aortic aneurysm rupture, thus regulating leukocyte trafficking and vascular inflammation [11]. Among RGS proteins, RGS4 has been widely studied in respiratory diseases. In severe asthma, RGS4, expressed in airway smooth muscle and the bronchial epithelium, was reported to promote allergen- and aspirin-associated airway hyperresponsiveness by inhibiting prostaglandin E2 biosynthesis [12]. In addition, RGS4 is a negative regulator of M(3) muscarinic receptors (M3Rs), and knockdown of RGS4 expression greatly enhanced the M3R-mediated increases in glucose-stimulated insulin secretion as well as calcium release in pancreatic beta cells [13]. RGS proteins also play a key role in numerous aspects of cancer, including tumorigenesis, progression, invasion and metastasis. RGS16 was reported to promote antitumor CD8^+^ T-cell exhaustion in an Erk1-dependent manner, thus affecting T-cell-based immunotherapies [14]. Upregulation of RGS1 in helper Th1 cells and cytotoxic T lymphocytes reduced their trafficking and survival in tumors, which was associated with shorter survival of patients with breast and lung cancer [15]. Additionally, RGS12 depletion promoted osteosarcoma progression and lung metastasis through the YAP-TEAD-Ezrin signaling pathway in an orthotopic xenograft mouse model [16].

RGS6, a member of the R7 subfamily, has two other domains in addition to the RGS domain: the disheveled EGL-10 and Pleckstrin homology (DEP) domain and the G gamma subunit-like (GGL) domain. These three domains coregulate the function, stability, and localization of the RGS6 protein [17]. In the past, RGS6 was widely studied in the nervous and cardiovascular systems. RGS6 has been implicated in several central nervous system pathologies associated with altered neurotransmission, including alcoholism, anxiety and depression, and Parkinson’s disease [17–20]. RGS6 was proven to negatively regulate GABA_B_ and D_2_ receptor-dependent inhibitory G protein signaling in dopaminergic neurons [21]. In association with G protein-coupled inwardly rectifying K^+^ channels, RGS6 is essential to regulate parasympathetic activation in the heart and functions to prevent parasympathetic override as well as severe bradycardia [22]. More commonly, RGS6 is regarded as an important target to modulate cancer risk in a variety of tumor types [23–27]. In the respiratory system, RGS6 was found to suppress TGF-β-induced epithelial–mesenchymal transition in vitro, and TGF-β was found to promote metastasis in vivo through canonical TGF-β-SMAD signaling in non-small cell lung cancer (NSCLC) [28]. An epidemiological study linking PDZ-RhoGEF polymorphisms with lung cancer risk suggested that there were interactions between RGS2, RGS6, and PDZ-RhoGEF and validated this family of proteins as key regulators of tumorigenesis. Recently, accumulating studies have demonstrated that RGS6 is involved in the occurrence and development of inflammatory diseases by regulating reactive oxygen species (ROS) production, apoptosis, and mitochondrial dysfunction [29, 30]. However, whether ALI/ARDS is influenced by RGS6 expression remains unknown. In this study, we examined the association between RGS6 and ALI/ARDS, hoping to contribute to identifying potential therapeutic targets for the clinical treatment of ARDS and increasing the cure rate for patients.

## Methods

### Mouse experimental protocol and tissue collection

Mice were housed in cages with easy access to food and water in a temperature- and humidity-controlled room with a 12-h dark/light cycle. RGS6^−/−^ mice were purchased from Cyagen Company, China. All mouse-related experiments were approved by the Animal Care and Use Committee of Fudan University (Y2021-425).

For the ALI model, 3 mg/kg lipopolysaccharide (LPS) (*Pseudomonas aeruginosa* 10, Sigma–Aldrich, USA) or *P. aeruginosa* (PA) reference strain PAO1 (4 × 10^5^ CFU) or an equal volume of PBS was injected intratracheally using a 24 G lavage needle after anesthetization by intraperitoneal injection of 25 mg/kg avertin (Sigma–Aldrich). Then, 0.2 ml of air was immediately injected, and the mouse was kept at 30° for 15 min. After waking up, the mice were placed back into their cages for feeding and were sacrificed on day 1 after PA (or PBS as control) administration or on day 3 after LPS (or PBS as control) administration.

For collection of bronchoalveolar lavage fluid (BALF), the right bronchus was ligated, and 0.5 ml PBS was lavaged through the trachea three times to obtain BALF. The BALF was centrifuged at 1500 rpm for 10 min. Then, the supernatant was collected and stored at − 80 ℃ for future analysis.

For hematoxylin–eosin (HE) staining, the right superior lobe was fixed with 4% paraformaldehyde (PFA) for 24 h, embedded in paraffin, sectioned at a thickness of 10 μm, and stained with HE. The pathological changes in mouse lung tissue were observed under a microscope after the sections were fixed and dried.

For immunohistochemical analysis, 3% hydrogen peroxide was dropped on the paraffin sections for 10 min, and citrate buffer was used for antigen retrieval. After goat serum blocking, the primary antibody was added and the sections were incubated for 12 h at 4 °C. The next day, the sections were washed three times with PBS and then incubated with the secondary antibody for 2 h. Finally, the sections were stained with 3,3′-diaminobenzidine (DAB) and sealed with resinene.

To measure the protein concentration in BALF, a BCA protein assay kit (Beyotime, China) was used according to the manufacturer’s instructions.

### RNA sequencing

Lung tissue was harvested from PBS control group mice and PAO1-infected group mice for RNA sequencing. Total RNA was extracted by TRIzol reagent (Invitrogen, USA) according to the manufacturer’s instructions. RNA purity and quantification were evaluated using a NanoDrop 2000 spectrophotometer (Thermo Fisher Scientific, USA). The GenSeq® rRNA Removal Kit (GenSeq, Inc.) was used to remove ribosomal RNA from samples. Libraries were constructed using the GenSeq® Low Input RNA Library Prep Kit (GenSeq, Inc.) according to the manufacturer’s instructions. The Bioanalyzer 2100 system (Agilent Technologies, USA) was used for quality control and quantification of constructed sequencing libraries. The libraries were sequenced on an Illumina NovaSeq 6000 platform, and 150 bp paired-end reads were generated. Raw reads in fastq format were first processed using fastp, and low-quality reads were removed to obtain clean reads. The clean reads were mapped to the mouse reference genome (UCSC MM10) using Hisat2 (v2.0.4). The FPKM3 of each gene was calculated, and the read counts of each gene were obtained by HTSeq-count. PCA was performed using R (v3.2.0) to evaluate the biological duplication of samples. Differential expression analysis was performed using DESeq2. A Q value < 0.05 and fold change > 2 or fold change < 0.5 were set as the thresholds for significantly differentially expressed genes (DEGs).

### Cell culture

RAW264.7 (ZQ0098) and MLE-12 (ZQ0470) cells were purchased from the Chinese Academy of Sciences (Shanghai, China). RAW264.7 cells were cultured in DMEM/high glucose medium (HyClone, USA) supplemented with 10% fetal bovine serum (FBS) (Gibco. USA) and 50 U/ml penicillin and streptomycin (Jinuo, China). MLE-12 cells were cultured in DMEM/F12 medium (HyClone, USA) supplemented with 2% FBS (Gibco. USA) and 50 U/ml penicillin and streptomycin (Jinuo, China). Cells were cultured at 37 °C in a 5% CO_2_ culture chamber. RAW264.7 cells were passaged through repeated pipetting with cold PBS. MLE-12 cells were passaged through trypsinization for 3 min at 37 °C. For RGS6 overexpression assays, the plasmid was purchased from Ribobio (China). Lipofectamine 3000 (Invitrogen, USA) was used to transfect plasmids into cells.

### Primary alveolar epithelial type II cells (AEC2s) isolation and culture

Primary AEC2s were isolated and cultured as described previously [31]. In brief, the mice were deeply anesthetized and exsanguinated through the abdominal aorta. The pulmonary circulatory system was perfused with normal saline via the pulmonary artery to remove excessive blood from the lung tissue. A 24 G lavage needle was inserted intratracheally, and the airways were lavaged with PBS to dislodge alveolar macrophages. Then, the alveolar epithelial cells were released by intratracheal administration of 0.125% pancreatin (Gibco, USA) and 0.5 mg/ml collagenase (Sigma, USA). The trachea was ligated after digestive enzyme administration, and lung tissue was removed from the thoracic cavity. The latter was placed into a sterile centrifuge tube at 37 ℃ in a water bath for 30 min. After mincing the lung tissue, 4% FBS and 100 mg/L deoxyribonuclease I (Sigma, China) were mixed with the lung tissue at 37 ℃ in a water bath for another 15 min. The liquid was repeatedly blown with pipettes to remove the tissue pieces. Then, the digestive fluid was sequentially filtered through 70- and 40-μm filters to obtain the cellular suspension. After centrifugation (300×g for 10 min), the cell pellet was resuspended in red blood cell lysis buffer (Beyotime, China) for 5 min to remove excess red blood cells. Then, DMEM/F12 medium was used to wash the cell pellet. The cellular suspension was purified by panning onto sterile plastic dishes coated with mouse IgG (Solarbio, China) for 1 h at 37 ℃. Nonadherent cells were centrifuged and resuspended in DMEM/F12 medium and then were transferred into sterile plastic dishes and incubated for 1 h at 37 ℃ another three times to remove endothelial cells and mesenchymal cells and obtain primary AEC2s for subsequent studies.

### Organoid culture

Organoid culture-related experiments were performed as described in previous studies [32]. In brief, primary AEC2s were resuspended in 10 mg/ml cold growth factor-reduced Matrigel (Corning, USA), and 20 μl drops of Matrigel-cell suspension were allowed to solidify on prewarmed 48-well suspension culture plates at 37 °C for 10–20 min. Upon complete gelation, 200 μl of alveolar organoid medium was added to each well, and plates were transferred to humidified 37 °C, 5% CO_2_ incubators at ambient O_2_ levels.

### Quantitative real-time PCR (qRT‒PCR)

Total RNA was extracted by TRIzol (Invitrogen, USA) according to the manufacturer’s instructions, and 1 μg of RNA was used for reverse transcription with Hifair III 1^st^ Strand cDNA Synthesis SuperMix (Yeasen, China). qRT–PCR was performed using Hieff qPCR SYBR Green Master Mix (Yeasen, China) according to the manufacturer’s instructions. The primer sequences are shown in Table 1. The relative mRNA expression level of each target gene was calculated by the 2^−△△CT^ method.

**Table 1:** Primers used in this study

Genes	Forward	Reverse
β-actin	CTACCTCATGAAGATCCTGACC	CACAGCTTCTCTTTGATGTCAC
RGS6	CAGCCAGCAGCGAGTGAAGC	GATCTTGGACAGACAGCCAGAACC
IL-1β	CACTACAGGCTCCGAGATGAACAAC	TGTCGTTGCTTGGTTCTCCTTGTAC
IL-6	CTTCTTGGGACTGATGCTGGTGAC	TCTGTTGGGAGTGGTATCCTCTGTG
SFTPC	CTGAGATGGTCCTTGAGATGAG	TCATGATGTAGCAGTAGGTTCC
SCGB1A1	CTCATGGAATCAGAGTCTGGTT	GATTTTCTCCGTGAGCTTCATG
TNF-α	CACCACGCTCTTCTGTCTACTGAAC	AGATGATCTGAGTGTGAGGGTCTGG
iNOS	GCAGAGATTGGAGGCCTTGTG	GGGTTGTTGCTGAACTTCCAGTC
MCP-1	CACTCACCTGCTGCTACTCATTCAC	CTTCTTTGGGACACCTGCTGCTG
Bcl-2	CCGTCGTGACTTCGCAGAGATG	ATCCCTGAAGAGTTCCTCCACCAC

### Western blotting

Total protein was extracted using RIPA (Beyotime, China) buffer containing PMSF (Beyotime, China) and phosphatase inhibitors (Bimake, USA). The protein concentration was measured using a BCA protein assay kit (Beyotime, China) according to the manufacturer’s instructions. Identical amounts of proteins were subjected to SDS‒PAGE (Yamei, China) and transferred to PVDF membranes (Millipore, USA). The membranes were then blocked with 5% bovine serum albumin for 1 h and then incubated with different primary antibodies overnight at 4 °C. After washing three times in TBST, the PVDF membranes were incubated with anti-rabbit horseradish peroxidase-conjugated secondary antibodies (Beyotime, China) for 1 h at room temperature. Immunoreactive bands were detected using ECL reagents (NCM Biotech, China) on a Bio-Rad imaging system. Antibodies against RGS6, p-p65, Bax, Bcl-2, and caspase-3 were purchased from Abcam (USA). The Antibody against IL-1β was purchased from Cell Signaling Technology (USA).

### ROS measurement

RAW264.7 cells were loaded with DCFDA (Beyotime, China) in serum-free DMEM for 30 min at 37 °C in a 5% CO_2_ incubator after different treatments. Then, the cells were washed with PBS three times and transferred by pipetting into flow cytometry tubes. ROS levels were quantified by flow cytometry.

### Enzyme-linked immunosorbent assay (ELISA)

The concentration of IL-6, MCP-1 and IL-1β in BALF was measured using ELISA kits (MULTI Sciences, China) according to the manufacturer’s instructions.

### Apoptosis assay

To assess apoptosis, an Annexin V-FITC/PI Cell Apoptosis Detection Kit (Servicebio, China) was used according to the manufacturer’s instructions. The apoptosis rate was determined by flow cytometry (Aria III, USA). For the caspase-3 activity test, a SuperView™ 488 caspase-3 Assay Kit for Live Cells (Yeasen, China) was used according to the manufacturer’s instructions. Nuclei were stained with Hoechst 33342 (Servicebio, China).

### Immunofluorescence staining

The cells were fixed with 4% paraformaldehyde for 5 min and then permeabilized with 0.1% Triton X-100 for 2 min. Bovine serum albumin (BSA, 5%) was used for blocking. Then, anti-RGS6 (Santa Cruz, USA), anti-tubulin (Beyotime, China), anti-PDPN (Santa Cruz, USA) or anti-SFTPC (Abcam, USA) primary antibodies were used for incubation at 4 °C overnight after dilution according to the manufacturer’s instructions. After washing with PBS three times, the cells were incubated with fluorescently labeled secondary antibodies (Beyotime, China) at room temperature in the dark for 1 h and washed with PBS three times. DAPI (Beyotime, China) staining was performed for 5 min, and PBS was used for washing. The sections were blocked with anti-fluorescence quenching sealing liquid. A terminal deoxynucleotidyl transferase dUTP nick end labeling (TUNEL) assay (Servicebio, China) was performed to detect apoptosis in lung tissue according to the manufacturer’s protocol.

### Statistical analysis

The 708 GPCR-related genes were downloaded from the GeneCards database. GO enrichment analysis and KEGG pathway analysis were performed using the Metascape database. Min Enrichment ≥ 1.5, Min Overlap ≥ 3, and *P* < 0.01 were considered thresholds. SPSS was used for statistical analysis. The results are shown as the mean ± SEM. Differences between groups were assessed using one-way analysis of variance. Differences with *P* < 0.05 were considered significant.

## Results

### Functional enrichment analysis of congruously regulated GPCR-related genes and DEGs in the ALI model

**Fig. 1:**
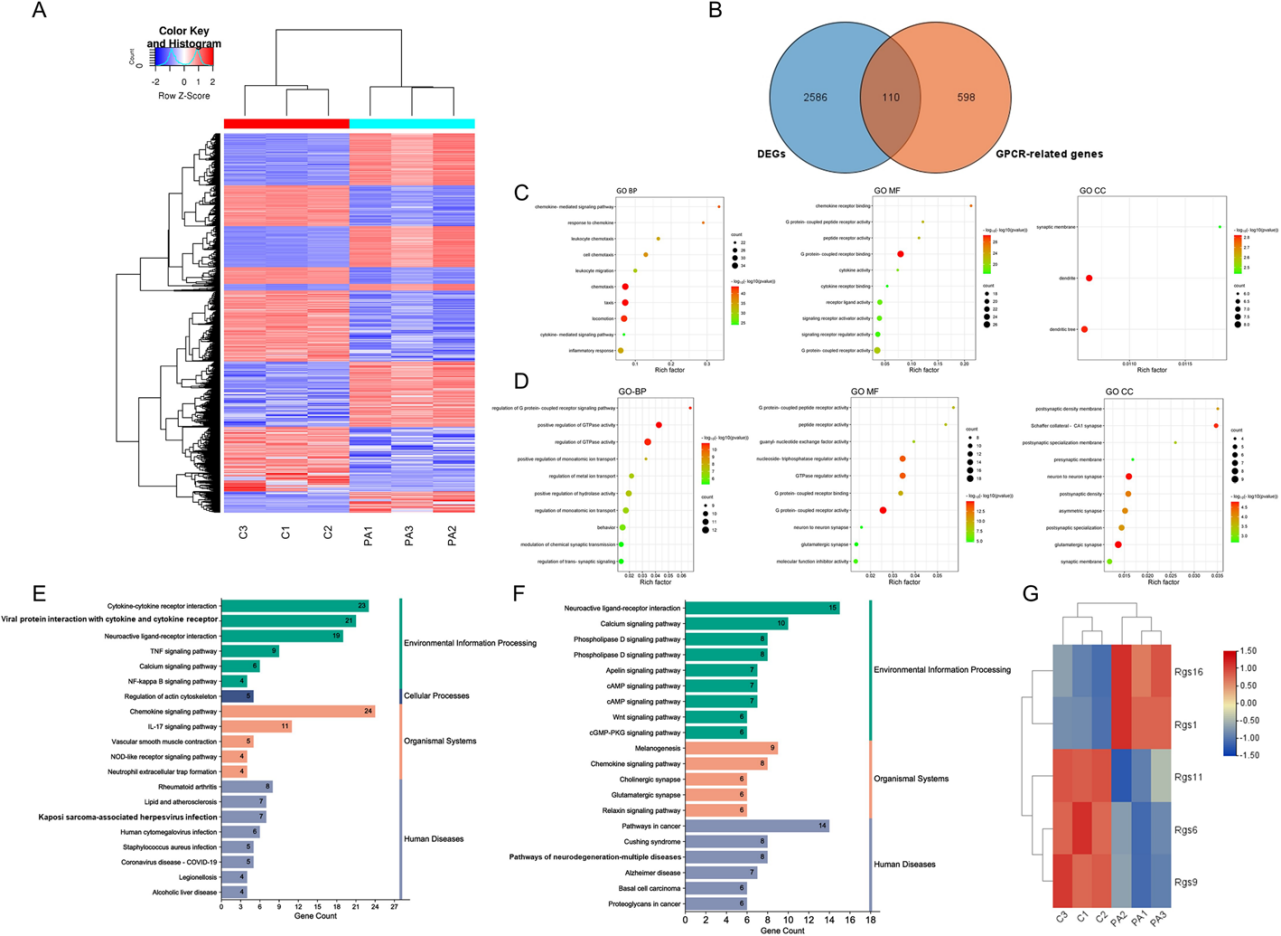
Functional enrichment analysis of congruously regulated GPCR-related genes and differentially expressed genes (DEGs) in the ALI model. **A** Heatmap showed the DEGs in *Pseudomonas aeruginosa* associated ALI model. **B** Veen diagram showed the congruously regulated genes in DEGs and GPCR-related genes. **C** The top 10 list of GO analysis for the 61 congruously upregulated genes. **D** The top 10 list of GO analysis for the 49 congruously downregulated genes. **E** The top 20 list of KEGG analysis for the 61 congruously upregulated genes. **F** The top 20 list of KEGG analysis for the 49 congruously downregulated genes. **G** Heatmap showed RGS family genes in these 110 congruously regulated genes

To establish the model of PA-associated ALI, the PA reference strain PAO1 was instilled intratracheally, and lung tissues were harvested from mice in the PBS and ALI groups for RNA sequencing. Differentially expressed genes (DEGs) were shown in Fig. 1A. The 708 GPCR-related genes were downloaded from the GeneCards database. Altogether, common genes identified as DEGs from lung tissue and GPCR-related genes were identified (Fig. 1B). We found 110 common genes in both DEGs and GPCR-related genes, including 61 congruously upregulated genes (up-CRGs) (Table 2) and 49 congruously downregulated genes (down-CRGs) (Table 3). Gene Ontology (GO) and Kyoto Encyclopedia of Genes and Genomes (KEGG) pathway analyses were performed to identify the biological functions of the common DEGs and their related pathways of enrichment. As shown in Fig. 1C, in the biological process (BP) category, the 61 up-CRGs were mostly involved in chemotaxis, taxis, locomotion and the inflammatory response. In the molecular function (MF) category, G protein-coupled receptor binding/activity, signaling receptor activator/binding activity and receptor ligand activity were the most enriched. In the cellular component (CC) category, the 61 up-CRGs were enriched in dendritic trees, synaptic membranes and dendrites. The 49 down-CRGs in the GO BP category were mostly involved in positive regulation of GTPase activity, positive regulation of hydrolase activity, and regulation of monoatomic ion transport and behavior. In the GO MF category, the main congruously downregulated genes were enriched in G protein-coupled receptor activity and GTPase regulator activity. In the GO CC category, glutamatergic synapse, neuron to neuron synapse and postsynaptic density were the most enriched terms (Fig. 1D). The results of KEGG pathway analysis of the 61 up-CRGs revealed that the most enriched pathways were the chemokine signaling, cytokine‒cytokine receptor interaction, and IL-17 and TNF signaling pathways (Fig. 1E). However, neuroactive ligand‒receptor interaction, pathways in cancer and the calcium signaling pathway were the pathways most strongly enriched in the 49 down-CRGs (Fig. 1F). Thus, inflammatory response-associated genes were enriched mostly in the up-CRGs, as evidenced by enrichment of chemokine receptors, which are a class of GPCRs that mediate the function of chemokines. The down-CRGs were closely related to calcium as well as cAMP signaling pathways, which have been extensively studied in the nervous and cardiovascular systems.

**Table 2:** 61 congruously upregulated genes in GPCR-related genes and DEGs in ALI model

Abr	Prok2	Ffar4	Anxa1	Ccr8	Hcrtr1
Grpr	Htr7	Adm	Bdkrb1	Ccr5	Ptafr
Gpr176	Htr4	Calca	Gpr84	Rgs1	C3
Ccl17	Calcb	Hrh2	Ccl7	Cxcr6	Cxcl10
Fgd3	Prokr1	Ccrl2	Ccl4	Mmp3	Cxcl16
Ccl11	Saa1	Fpr1	Ccl3	Cxcl9	Cxcl13
Ccl22	Vav1	Fpr3	Ccl2	Cxcl5	Rgs16
Ccl20	Edn1	Fpr2	Cxcr2	Cxcl3	Itga5
Adm2	Arrb2	Hbegf	Cxcr1	Cxcl2	Lpar1
Adora3	Ffar2	Gpr35	Ccr1	Cxcl1	Rhoc
Ackr3

**Table 3:** 49 congruously downregulated genes in GPCR-related genes and DEGs in ALI model

Gng8	Arhgef15	Gna14	Ednrb	Rgs6	Fzd7
Grm8	Adcy8	Fzd10	Ptgfr	Adrb3	Grp
Gpsm1	Adcy1	Ngef	Ccr3	Aplnr	Lpar3
Arhgef9	Cd55	Rasgrp2	Cckar	Ptch1	Dgkb
Apln	Edn3	Gpr17	Penk	Wnt2	Dgkg
Tiam2	Ramp1	Plcb1	Cxcr5	Wnt4	Gng11
Arhgef38	Rgsl1	Chrm1	Rgs9	Hcrtr2	Tshb
Trpc3	Ffar1	Rasgrf2	Adrb1	Fzd3	Ackr4
Rgs11					

We analyzed RGS family genes in these 110 CRGs and found that RGS16 and RGS1 were upregulated and that RGS11, RGS6 and RGS9 were downregulated (Fig. 1G). Considering that RGS6 was the only protein with G protein-dependent and -independent functions and was also closely connected with inflammation, we then studied the key role of RGS6 in ALI models.

### RGS6 was downregulated in ALI models in vivo and in vitro

**Fig. 2:**
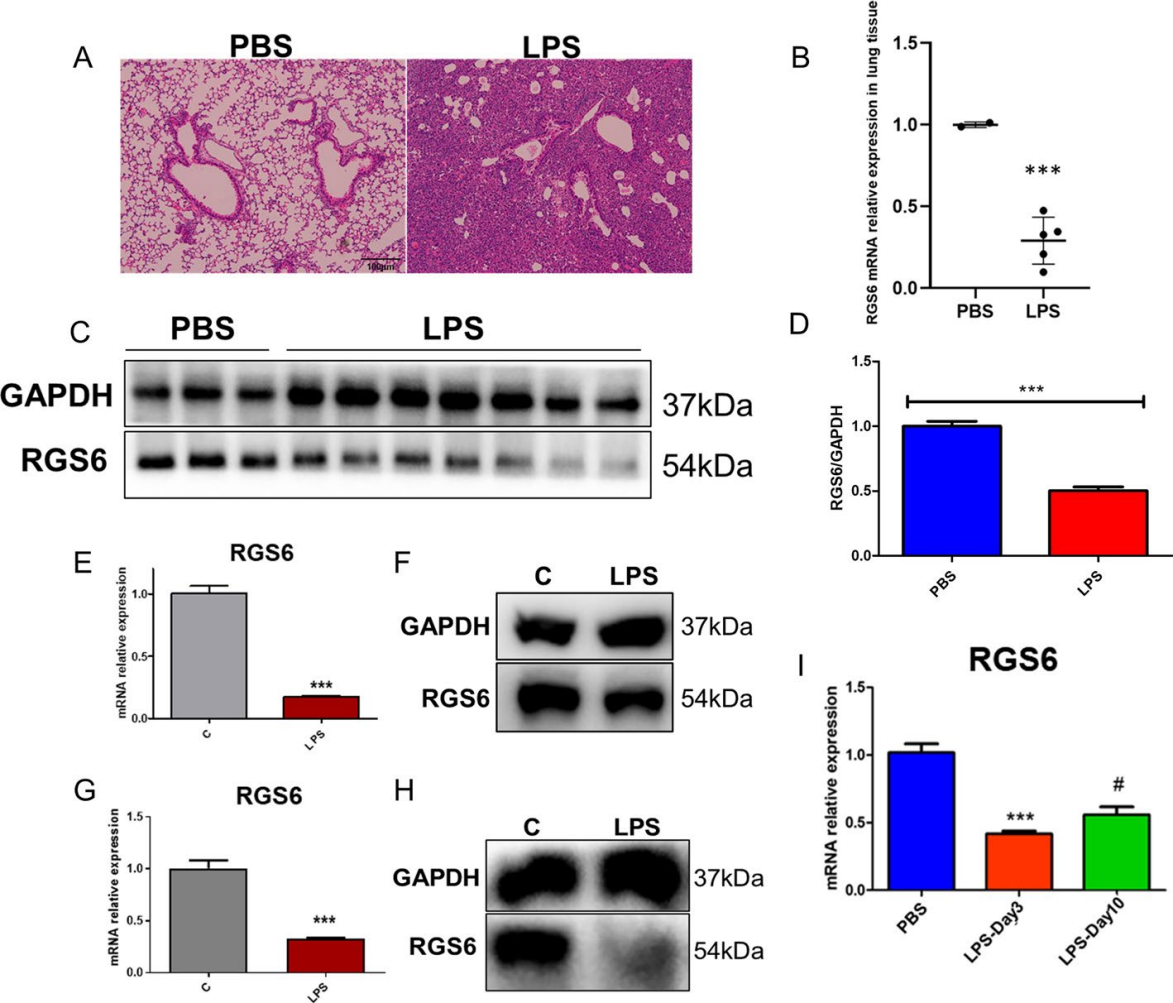
RGS6 was downregulated in ALI models in vivo and in vitro. **A** HE staining showed pathological changes in lung tissue after LPS administration. Scale bar: 100 μm. **B** RGS6 mRNA relative expression levels in lung tissue after LPS administration. **C** Western blotting showed RGS6 protein expression in lung tissue after LPS administration. **D** Quantitative analysis showed RGS6 protein relative expression levels in lung tissue in PBS and LPS group. E. RGS6 mRNA relative expression levels in RAW264.7 cells after LPS administration (100 ng/ml for 8 h). **F** Western blotting showed RGS6 protein expression levels in RAW264.7 cells after LPS administration. **G** RGS6 mRNA relative expression levels in MLE-12 cells after LPS administration (1ug/ml for 24 h). **H** Western blotting showed RGS6 protein expression levels in MLE-12 cells after LPS administration. **I** RGS6 mRNA relative expression levels in lung tissue 3 days and 10 days after LPS administration. Data are shown as mean ± SEM. PBS, wild type mice given PBS intratracheally and executed at day 3. LPS, wild type mice given LPS (5 mg/kg) intratracheally and executed at day 3. C: cells without LPS stimulation. LPS: cells with LPS stimulation. ^***^P < 0.001 vs. PBS group or C group; ^#^*P* < 0.05 vs. LPS-Day 3 group

We then used intratracheal administration of *P. aeruginosa*-derived LPS to construct an ALI model. As shown in Fig. 2A, the pathological changes in mouse lung tissue in the ALI group included extensive inflammatory cell infiltration, thickened alveolar walls and pulmonary consolidation compared with those in the PBS control group. The mice developed respiratory distress and weight loss after LPS administration. We assessed the dynamic change in the RGS6 expression level in lung tissue of the ALI model on day 3, during which time the inflammatory damage was determined to be the highest. qRT‒PCR and western blotting analyses revealed that RGS6 was downregulated in the lung tissue of the ALI model at both the mRNA and protein levels (Fig. 2B–D). The hypothesis that cytokine storm results in an uncontrolled inflammatory response is a widely accepted pathophysiological mechanism in ARDS [33]. Macrophages play an important role in triggering cytokine storm. In addition, the alveolar epithelium forms a blood-gas barrier and responds to many foreign substances. Thus, we measured RGS6 expression levels in the macrophage cell line RAW264.7 and the alveolar epithelial cell line MLE-12 after LPS stimulation. We found that RGS6 was significantly downregulated in RAW264.7 cells after LPS stimulation (Fig. 2E and 2F) and in MLE-12 cells (Fig. 2G and 2H). Interestingly, RGS6 expression was observed to be upregulated in lung tissue 10 days after LPS administration in vivo compared to that on day 2, during which time the pulmonary tissue retained self-regeneration ability and lung tissue began to undergo repair (Fig. 2I).

### RGS6 knockout aggravated acute lung injury in mice

**Fig. 3:**
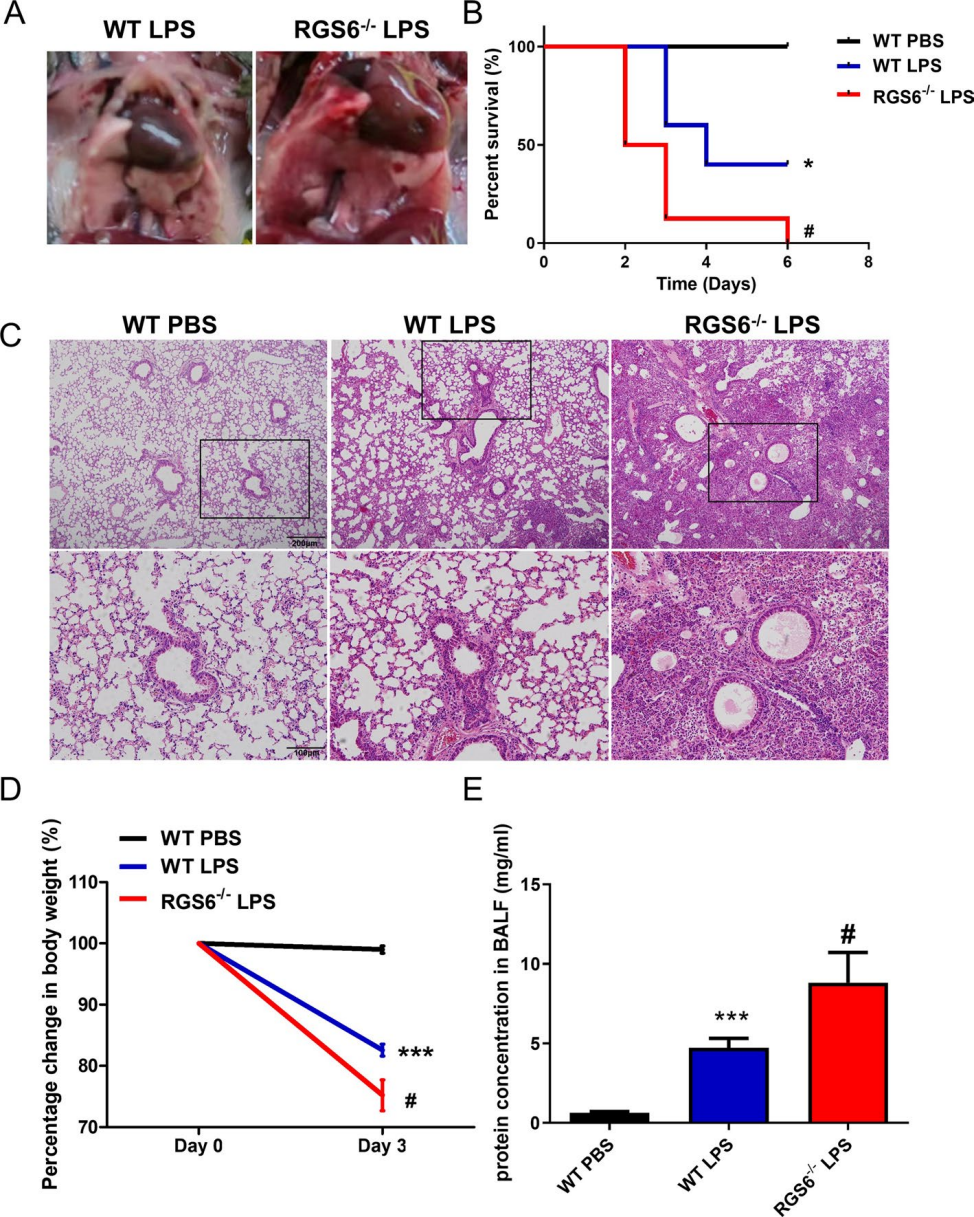
Acute lung injury was aggravated upon RGS6 knockout. **A** General observation of the change of lung tissue. **B** Kaplan–Meier curve showed survival of mice in different groups. **C** HE staining showed pathological changes in different groups. Scale bar: upper 200 μm, lower 100 μm. **D** Percentage change of body weight of mice in different groups. **E** Protein concentration in BALF in different groups. WT PBS, wild type mice given PBS intratracheally and executed at day 3; WT LPS, wild type mice given LPS (3 mg/kg) intratracheally and executed at day 3; RGS6^−/−^ LPS, RGS6^−/−^ mice given LPS (3 mg/kg) intratracheally and executed at day 3. Data are shown as mean ± SEM. ^*^P < 0.05 vs. WT PBS group; ^***^P < 0.001 vs. WT PBS group; ^#^*P* < 0.05 vs. WT LPS group

RGS6 knockout mice were used to study RGS6 function in ALI. LPS (3 mg/kg) derived from PA was administered intratracheally into mouse lungs to construct the ALI model. The overall survival curve showed that the RGS6 knockout mice in the LPS group died earlier and the mortality rate was significantly higher than that of the wild-type (WT) LPS group (Fig. 3B). Additionally, weight loss was more pronounced in the RGS6^−/−^ LPS group (Fig. 3D). Although the lesions in the mouse lungs of the ALI group were inhomogeneous, the lesions were larger and more extensive upon RGS6 knockout (Fig. 3A). HE staining showed that inflammatory cell infiltration was more obvious and the consolidation lesion area of lung tissue was more extensive in the RGS6^−/−^ LPS group than in the WT LPS group (Fig. 3C). However, no significant difference in histological structures was observed between WT and RGS6^−/−^ mice without LPS administration (Additional file 1: Fig. S1A). In addition, we also determined the total protein concentration in BALF to evaluate the integrity of the air-blood barrier function. We found that protein concentration in BALF was higher at baseline and the impairment of air-blood barrier function induced by LPS was greater upon RGS6 knockout (Fig. 3E, Additional file 1: Fig. S1B). Thus, RGS6 knockout aggravated ALI, indicating that RGS6 may function as a protective factor in the occurrence and progression of ALI.

### RGS6 knockout promoted inflammatory cell accumulation and apoptosis in the lungs of ALI models

**Fig. 4:**
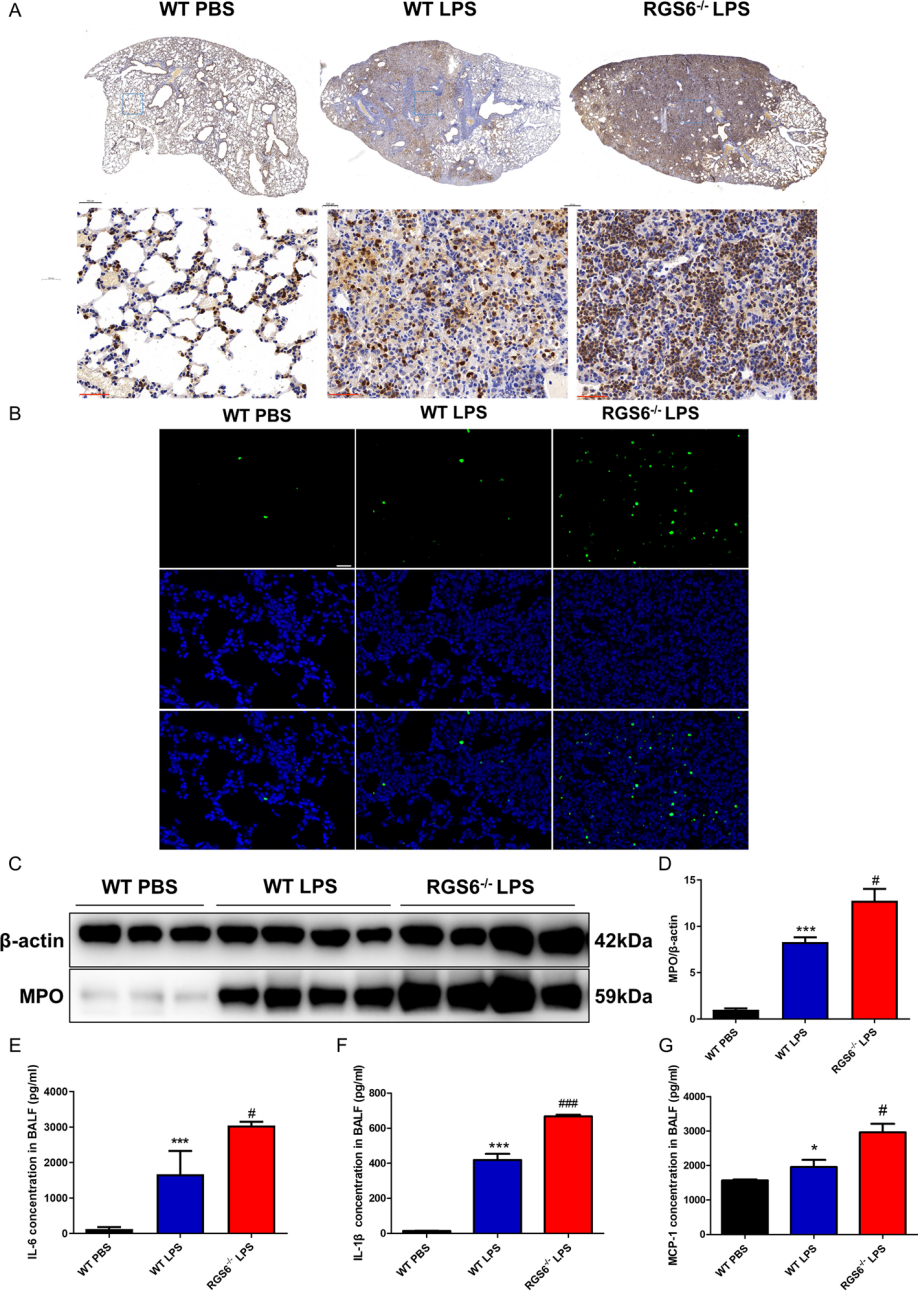
RGS6 knockout promoted inflammation and apoptosis in the lungs of ALI models. **A** Immunohistochemistry staining of Ly6g showed neutrophils infiltration in different groups. Scale bar: black 500 μm, red 50 μm. **B** Tunnel staining showed cell apoptosis in different groups. Cell nucleus was stained by DAPI (blue fluorescence). Apoptotic cells were stained by TUNEL (green fluorescence). Scale bar: white 50 μm. **C** Western blotting showed MPO expression levels of lung tissue in different groups. **D** Quantitative analysis for MPO relative protein expression levels in lung tissue. **E** ELISA detected IL-6 concentration in BALF in different groups. **F** ELISA detected IL-1β concentration in BALF in different groups. **G** ELISA detected MCP-1 concentration in BALF in different groups. WT PBS, wild type mice given PBS intratracheally and executed at day 3; WT LPS, wild type mice given LPS (3 mg/kg) intratracheally and executed at day 3; RGS6^−/−^ LPS, RGS6^−/−^ mice given LPS (3 mg/kg) intratracheally and executed at day 3. Data are shown as mean ± SEM. ^*^P < 0.05 vs. WT PBS group; ^***^P < 0.001 vs. WT PBS group; ^#^*P* < 0.05 vs. WT LPS group; ^###^*P* < 0.001 vs. WT LPS group

To assess whether RGS6 influenced the inflammation level in ALI, we used immunohistochemical staining to evaluate Ly6g-positive cells, a representative marker of neutrophils, in the lung tissue of each group. We found that many neutrophils accumulated in the lungs after LPS administration. No significant difference was found without LPS administration between WT and RGS6^−/−^ mice (Additional file 1: Fig. S1F). However, the RGS6^−/−^ LPS mice showed much more neutrophil accumulation than the WT LPS group (Fig. 4A). TUNEL staining revealed that LPS administration promoted apoptosis, and the level of apoptosis in lung tissue increased upon RGS6 knockout (Fig. 4B). In addition, relative myeloperoxidase (MPO) expression in lung tissue was determined by western blotting. We found that LPS administration significantly promoted MPO upregulation in lung tissue, especially in RGS6 knockout mice (Fig. 4C, 4D). The concentration of proinflammatory factors IL-6, IL-1β and MCP-1 in BALF was much higher in the RGS6^−/−^ LPS group than in the WT LPS group (Fig. 4E–G). We also detected the concentration of IL-6, IL-1β and MCP-1 in BALF at baseline, which showed higher levels in RGS6^−/−^ mice than in WT mice except for MCP-1 (Additional file 1: Fig. S1C–E). RGS6 knockout promoted inflammation and apoptosis in the progression of ALI.

### RGS6 knockout reduced the regenerative capacity of AEC2s

**Fig. 5:**
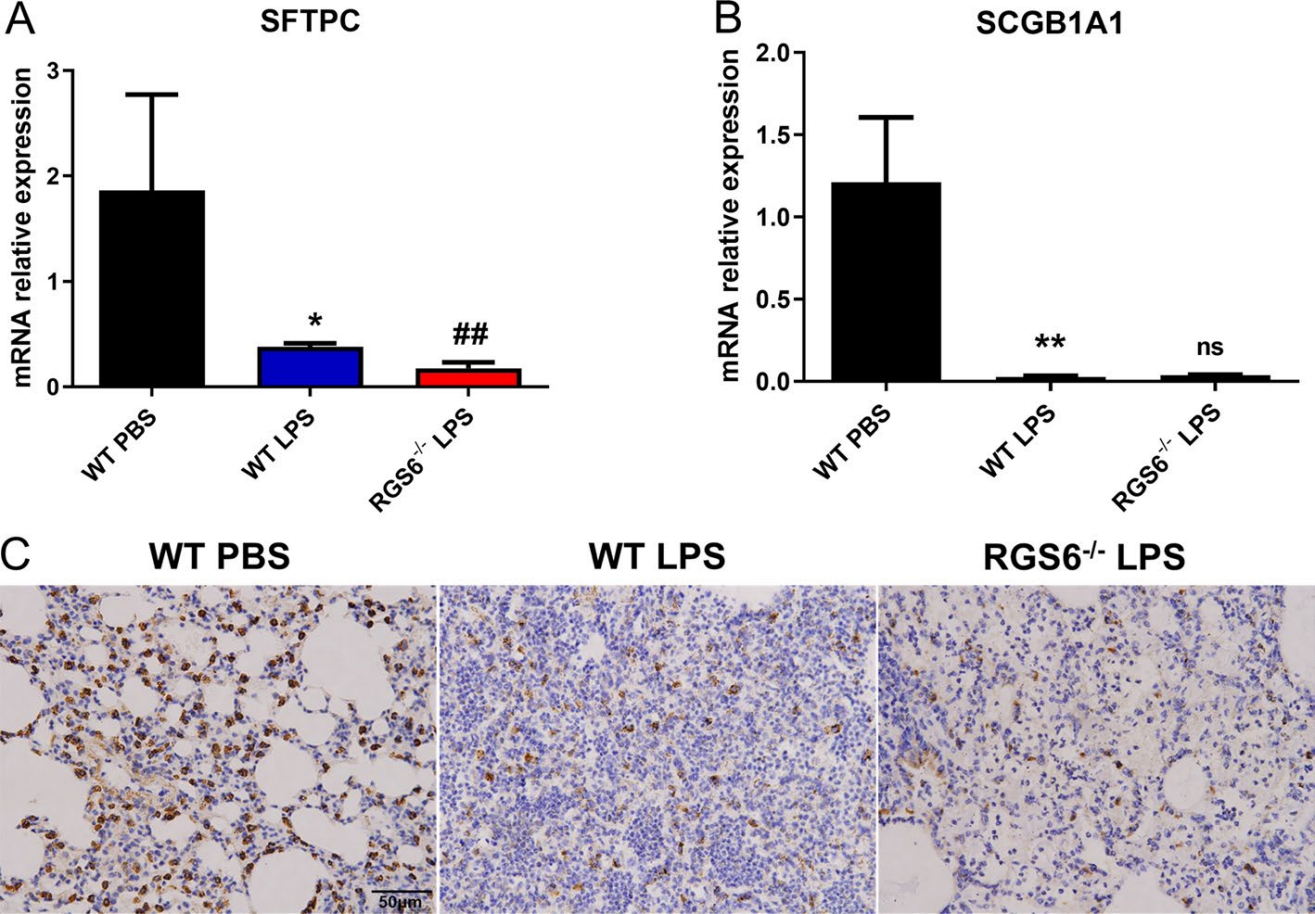
RGS6 knockout reduced the regenerative capacity of AEC2s. **A** qRT-PCR showed SFTPC mRNA relative expression levels in lung tissue in different groups. **B** qRT-PCR showed SCGB1A1 mRNA relative expression levels in lung tissue in different groups. **C** Immunohistochemistry showed AEC2 loss in different groups. AEC2s were stained with anti-SFTPC antibody. Scale bar: 50 μm. WT PBS, wild type mice given PBS intratracheally and executed at day 3; WT LPS, wild type mice given LPS (3 mg/kg) intratracheally and executed at day 3; RGS6^−/−^ LPS, RGS6^−/−^ mice given LPS (3 mg/kg) intratracheally and executed at day 3. Data are shown as mean ± SEM. ^*^P < 0.05 vs. WT PBS group; ^**^P < 0.01 vs. WT PBS group ^##^*P* < 0.01 vs. WT LPS group. ^ns^, no significance vs. WT LPS group

Lung regeneration mainly relies on endogenous stem cells to reconstruct alveolar structure and function. AEC2s and club cells are important somatic stem cells that participate in alveolar and bronchial repair [34, 35]. We detected the representative marker of AEC2s (SFTPC) and club cells (SCGB1A1) in lung tissue. We found that an overactive inflammatory response triggered by LPS seriously damaged structural cells in the lung. Compared to the WT LPS group, the AEC2 marker (SFTPC) was more severely damaged upon RGS6 knockout (Fig. 5A, 5B). The immunohistochemical staining results also revealed that AEC2 loss was more pronounced in the RGS6^−/−^ LPS group (Fig. 5C).

### RGS6 knockout reduced the stemness and self-renewal capacity of AEC2s

**Fig. 6:**
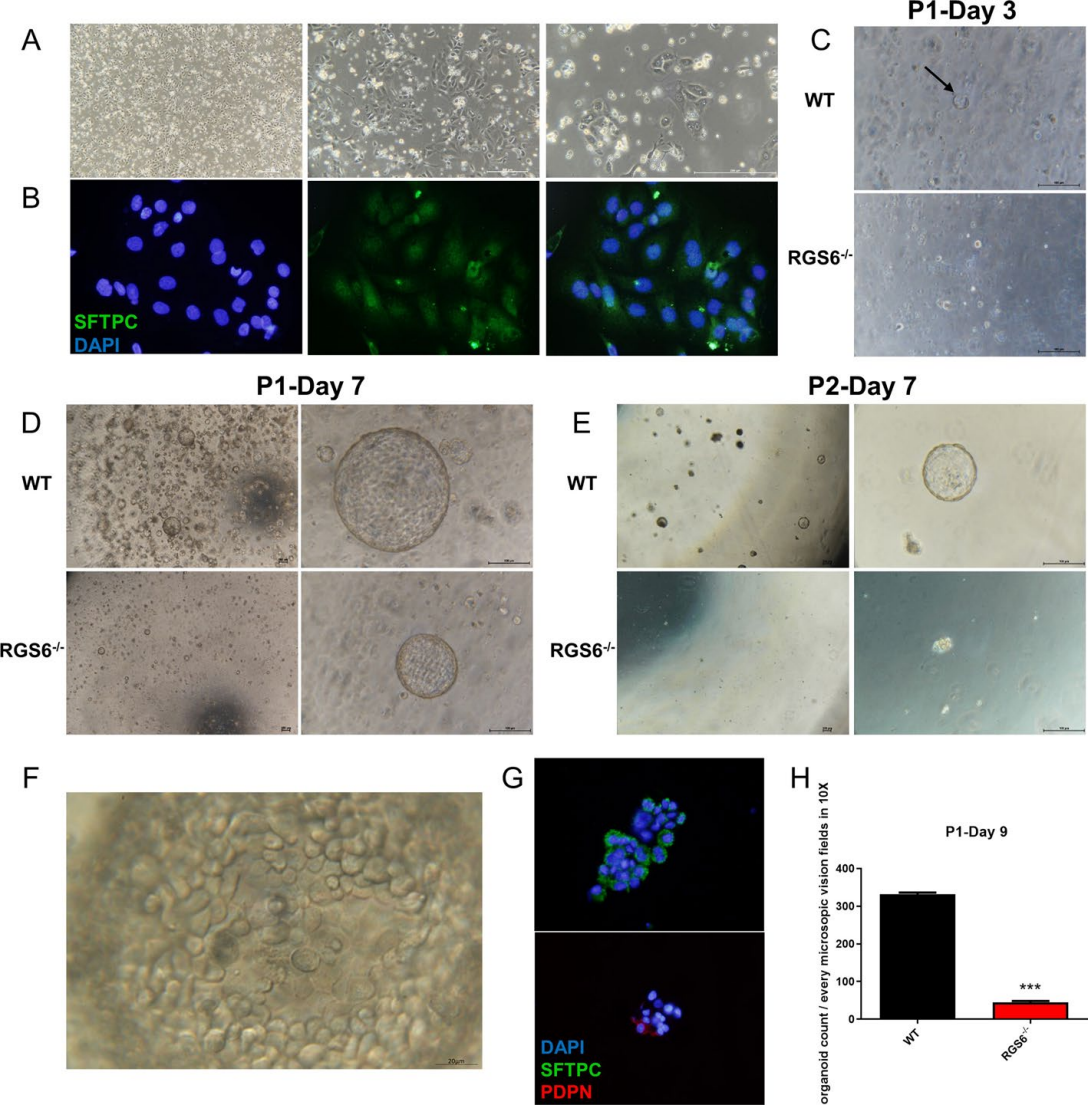
RGS6 knockout reduced the stemness and self-renewal capacity of AEC2s. **A** Primary AEC2s morphology was observed under light microscope. **B** Immunofluorescence staining showed the purity of AEC2s. Cell nucleus was stained by DAPI (blue fluorescence). AEC2s were stained by anti-SFTPC antibody (green fluorescence). **C** Organoids came to being at day 3, characterized as two or three cells grouped to form a ring structure (as shown by the black arrow). Alveolar organoids formation from RGS6^−/−^ mice was slower compared to that from WT mice. **D** Alveolar organoids observation at day 7. **E** Alveolar organoids could still form and develop after passaging in WT group, which was rarely seen in RGS6^−/−^ group. **F** The internal structure of alveolar organoids was shown under high magnification microscope. **G** Immunofluorescence staining showed AEC2s could differentiate into AEC1s in alveolar organoids. Cell nucleus was stained by DAPI (blue fluorescence). AEC2s were stained by anti-SFTPC antibody (green fluorescence). AEC1s were stained by anti-PDPN antibody (red fluorescence). **H** Alveolar organoids counting at day 9 in different groups. Scale bars were shown in the pictures. WT, the primary AEC2s isolated from lung tissue of wild type mice and cultured in Matrigel. RGS6^−/−^, the primary AEC2s isolated from lung tissue of RGS6^−/−^ mice and cultured in Matrigel. Data are shown as mean ± SEM. ^***^P < 0.001 vs WT group

To study whether RGS6 influenced the biological function of AEC2s, we cultured primary AEC2s using differential adhesion methods as described previously [31]. As shown in Fig. 6A, the morphology of primary AEC2s looked like paving stones. Immunofluorescence staining showed that these cells expressed SFTPC, the specific marker of AEC2s (Fig. 6B). In this case, we purified and cultured primary AEC2s from WT and RGS6^−/−^ mice. After adjusting the cell density, we resuspended primary AEC2s in Matrigel to perform organoid culture. We found that WT alveolar organoids began to form on day 3 due to the stemness of AEC2s and their self-assembly capacity. However, RGS6^−/−^ alveolar organoids formed much later (Fig. 6C). On day 7, we observed abundant WT alveolar organoid formation in the wells. RGS6^−/−^ alveolar organoids were less abundant and smaller in volume than WT alveolar organoids (Fig. 6D). We counted the number of alveolar organoids in each group at day 9 and found that their quantity significantly decreased upon RGS6 knockout (Fig. 6H). Under high magnification with a microscope, protuberance structures similar to alveoli were observed inside the organoid (Fig. 6F). Immunofluorescence staining revealed that alveolar organoids were mainly composed of AEC2s and alveolar epithelial type I cells (AEC1s) (Fig. 6G). We passaged the alveolar organoids and found that the cells derived from WT mice could form new organoids due to their self-assembly capacity. However, cells derived from RGS6^−/−^ mice had difficulty forming new organoids (Fig. 6E). Therefore, RGS6 may play an important role in the stemness, self-renewal and self-assembly capacity of AEC2s.

### Overexpression of RGS6 decreased ROS production and inflammatory cytokine transcription in RAW264.7 cells

**Fig. 7:**
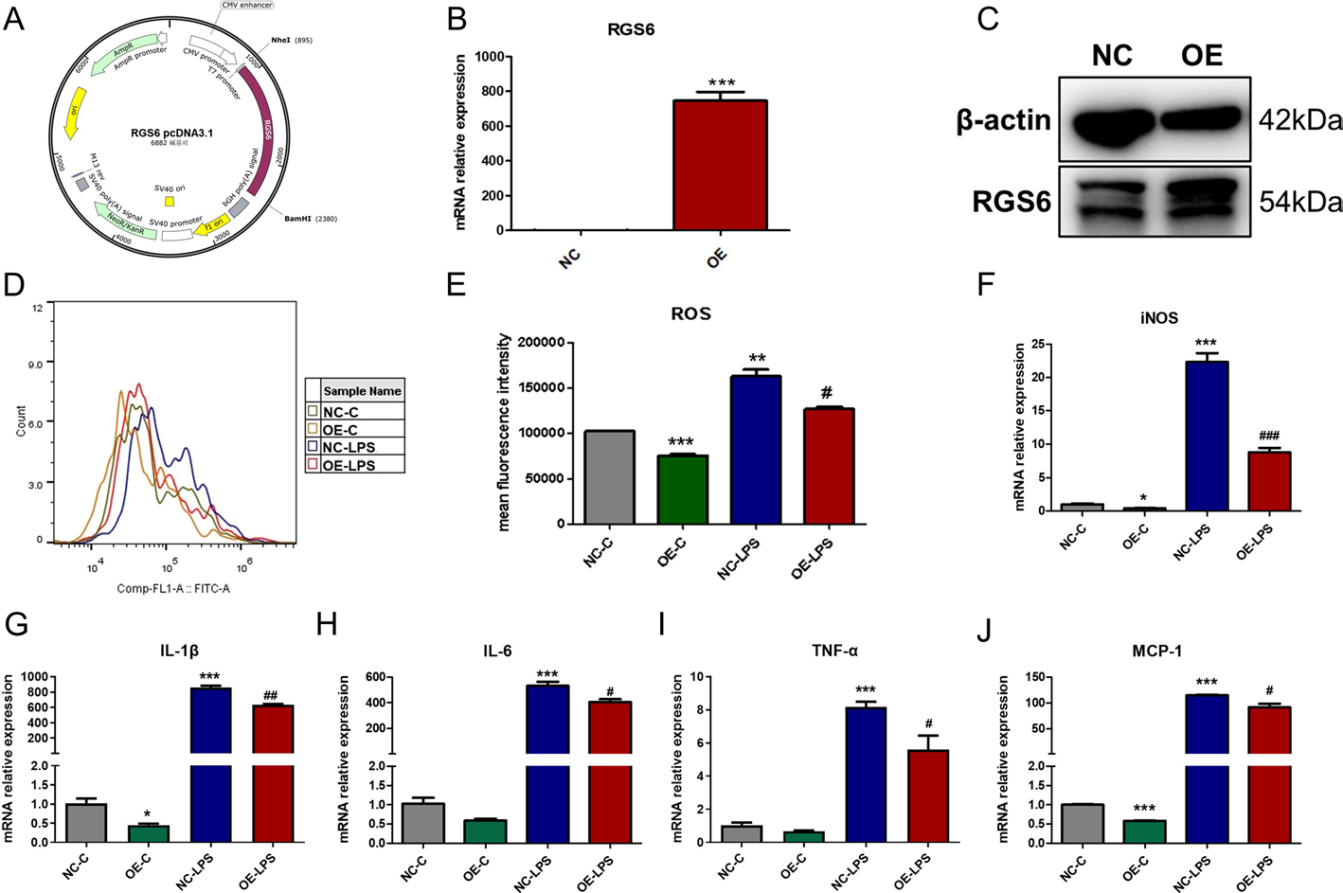
RGS6 overexpression in macrophage decreased intracellular ROS production and proinflammatory cytokine transcription. **A** Structural map of RGS6 pcDNA3.1(+). **B** RGS6 overexpression efficiency was shown by qRT-PCR. Control group was flagged as NC, while RGS6 overexpression group was flagged as OE. **C** Western blotting showed overexpression of RGS6 protein in RAW264.7 cells. **D** Flow cytometry showed intracellular ROS production in RAW264.7 cells. **E** Quantitative analysis showed mean fluorescence intensity about DCFH-DA-FITC in RAW264.7 cells. Proinflammatory cytokines iNOS (**F**), IL-1β (**G**), IL-6 (**H**), TNF-α (**I**) and MCP-1 (**J**) were detected by qRT-PCR. NC-C, RAW264.7 cells transfected with control plasmid for 24 h followed by culturing for another 8 h. OE-C, RAW264.7 cells transfected with RGS6 overexpression plasmid for 24 h followed by culturing for another 8 h. NC-LPS, RAW264.7 cells transfected with control plasmid for 24 h followed by stimulating with LPS (100 ng/ml) for another 8 h. OE-LPS, RAW264.7 cells transfected with RGS6 overexpression plasmid for 24 h followed by stimulating with LPS (100 ng/ml) for another 8 h. Data are shown as mean ± SEM. *P < 0.05 vs. NC-C group; **P < 0.01 vs. NC-C group; ***P < 0.001 vs. NC-C group. ^#^*P* < 0.05 vs. NC-LPS group; ^##^*P* < 0.01 vs. NC-LPS group. ^###^*P* < 0.001 vs. NC-LPS group

To study the role of RGS6 in inflammation, we constructed a plasmid to overexpress RGS6 in RAW264.7 cells. The structure of the plasmid is shown in Fig. 7A. The overexpression efficacy is shown in Fig. 7B and 7C. We found that RGS6 overexpression significantly reduced the level of intracellular ROS (Fig. 7D, E) as well as the iNOS transcriptional level (Fig. 7F). In addition, RGS6 overexpression decreased the transcription of proinflammatory cytokines, such as IL-1β, IL-6, TNF-α and MCP-1 (Fig. 7G–J).

### RGS6 overexpression reduced apoptosis in MLE-12 cells

**Fig. 8:**
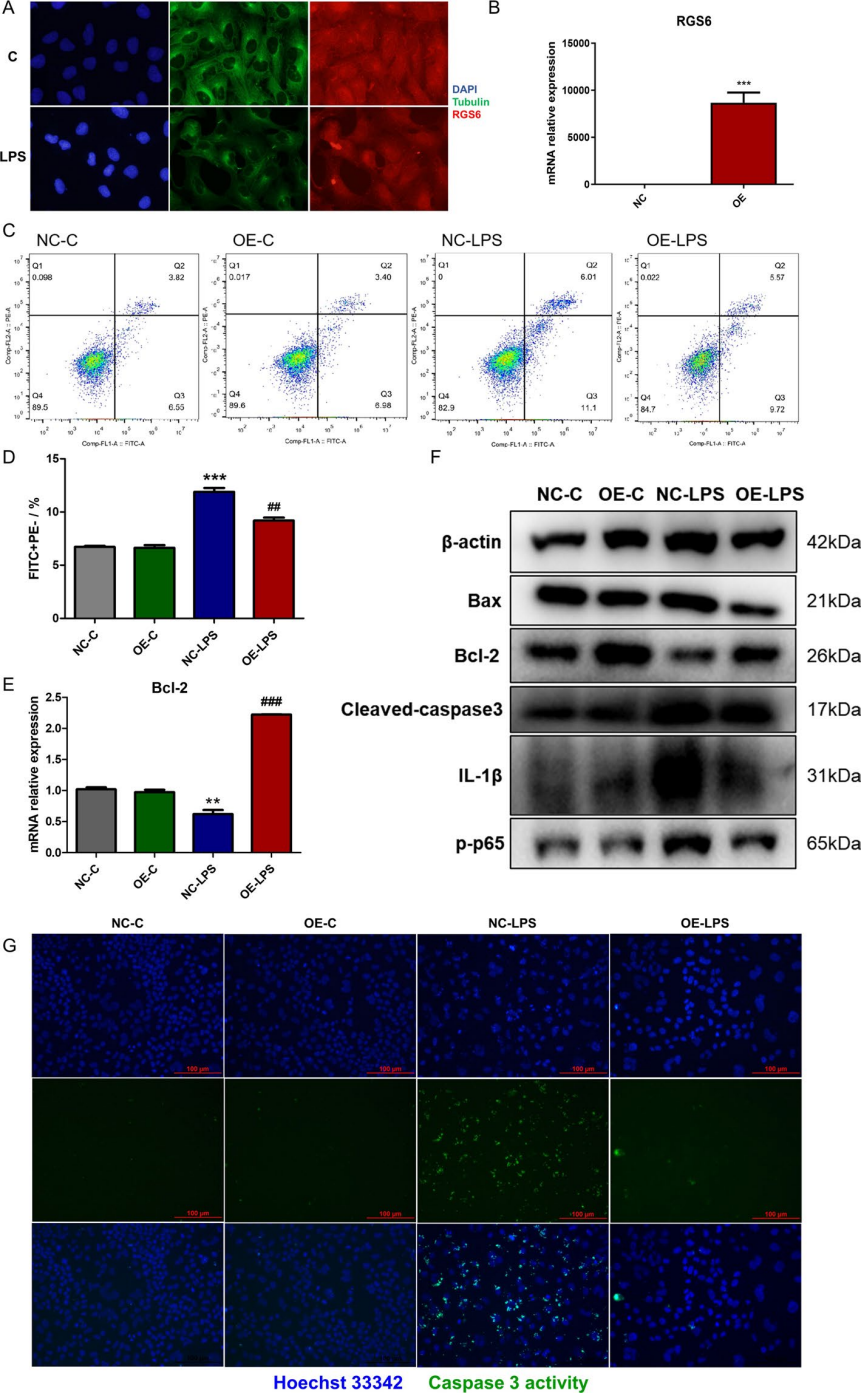
RGS6 overexpression reduced apoptosis in MLE-12 cells. **A** Immunofluorescent staining showed RGS6 positional changes after LPS stimulation. Cell nucleus was stained by DAPI (blue fluorescence). Cytoplasm was stained by anti-β Tubulin antibody (green fluorescence). RGS6 protein was stained by anti-RGS6 antibody (red fluorescence). Scale bar: 20 μm. **B** RGS6 overexpression efficiency in MLE-12 cells was shown by qRT-PCR. Control group was flagged as NC, while RGS6 overexpression group was flagged as OE. **C** Flow cytometry showed Annexin V-FITC/PI changes in different groups. **D** Quantitative analysis showed the percentage of early apoptotic cells (FITC + PE−). **E** Bcl-2 mRNA relative expression levels in different groups. **F** Western blotting showed protein expression levels in different groups. **G** Caspase 3 activity assays for live cells in different groups. Cell nucleus was stained by Hoechst 33342 (blue fluorescence). Scale bar: 100 μm. NC-C, MLE-12 cells transfected with control plasmid for 24 h followed by culturing for another 24 h. OE-C, MLE-12 cells transfected with RGS6 overexpression plasmid for 24 h followed by culturing for another 24 h. NC-LPS, MLE-12 cells transfected with control plasmid for 24 h followed by stimulating with LPS (1 μg/ml) for another 24 h. OE-LPS, MLE-12 cells transfected with RGS6 overexpression plasmid for 24 h followed by stimulating with LPS (1 μg/ml) for another 24 h. Data are shown as mean ± SEM of triplicate. *P < 0.05 vs. NC-C group; **P < 0.01 vs. NC-C group; ***P < 0.001 vs. NC-C group. ^#^*P* < 0.05 vs. NC-LPS group; ^##^*P* < 0.01 vs. NC-LPS group. ^###^*P* < 0.001 vs. NC-LPS group

Immunofluorescence staining was performed to determine RGS6 positional changes in MLE-12 cells. We found that RGS6 was mainly expressed in the cytoplasm. RGS6 protein accumulation in the nucleus increased after LPS stimulation (Fig. 8A). We also overexpressed RGS6 in the mouse lung epithelial cell line MLE-12 to determine its impact on apoptosis (Fig. 8B). Annexin-V/PI staining showed that RGS6 overexpression reduced the proportion of early apoptotic MLE-12 cells upon LPS stimulation (Fig. 8C, 8D). In addition, we observed downregulation of the proapoptotic protein Bax and upregulation of the antiapoptotic protein Bcl-2 following RGS6 overexpression compared to the NC-LPS group (Fig. 8E, 8F). Cleaved caspase-3 expression was downregulated upon RGS6 overexpression compared to that in the NC-LPS group (Fig. 8C). Caspase-3 activity in live cells was also assessed in different groups. The substrate was initially nonfluorescent and passed through the cell membrane into the cytoplasm. In apoptotic cells, caspase-3/7 cleaved the substrate to release a high-affinity DNA dye, which migrated into the nucleus to label DNA, in which case bright green fluorescence was observed. Inhibition control revealed that no caspase-3 activity was detected while pretreated by caspase-3 inhibitor Ac-DEVD-CHO (Additional file 1: Fig. S2). LPS stimulation resulted in elevated caspase-3 activity in MLE-12 cells. However, RGS6 overexpression inhibited caspase-3 activity and apoptosis (Fig. 8G). We also found that RGS6 overexpression inhibited the NF-κB signaling pathway as well as the expression of the proinflammatory cytokine IL-1β (Fig. 8F). Therefore, RGS6 may function to protect the lung epithelium against apoptosis during the progression of ALI.

## Discussion

ARDS/ALI is a critical clinical syndrome characterized by an uncontrolled cytokine storm and impaired regenerative capacity of endogenous stem cells. How to control the inflammatory response effectively at an early stage and simultaneously mobilize endogenous stem cells to accelerate regeneration are the key scientific concerns in the treatment of ALI/ARDS. The pathological process of ARDS is very complicated. GPCR family proteins have attracted extensive attention due to their wide range of targets and clear influence on every physiological process.

Accumulating studies have revealed that GPCR family proteins play a non-negligible role in the occurrence and progression of ALI/ARDS, especially in the cytokine storm during the acute phase of the disease [36–39]. Neutrophils, one of the most important components in the cytokine storm, must be simultaneously activated by integrin and GPCR signaling to induce neutrophil extracellular trap formation in ALI, which is associated with the high mortality in critically ill patients [36]. GPR4, a proinflammatory factor highly expressed in vascular endothelial cells, was able to regulate vascular permeability, leukocyte infiltration and tissue edema under inflammatory conditions [37]. GPCR family proteins also participate in the reconstruction phase after lung injury. AEC2s can self-renew and differentiate for long periods. Specifically, ablated AEC2s cause individual survivors to undergo rapid clonal expansion and daughter cell dispersal [35]. Thus, AEC2s are widely recognized as the main driver of lung regeneration after ALI/ARDS. Epithelial GPR116 was reported to control surfactant secretion and reuptake in AEC2s by regulating Gq/11 signaling, which regulates pulmonary alveolar homeostasis [40]. In addition, GPCR family proteins also promote lung regeneration by targeting club cells [41].

Considering the important effects of RGS family proteins on GPCRs, we identified 110 congruously regulated GPCR-related genes and differentially expressed genes in an ALI model in this study. Five RGS genes were identified, namely, 2 that were upregulated and 3 that were downregulated (Fig. 1). Based on the described close relationship between RGS6 and inflammation in the literature, we then focused on RGS6 and studied whether it can influence ALI/ARDS.

RGS6, a member of the RGS protein family, acts as a GTPase-activating protein to accelerate the intrinsic GTPase activity of Gα, resulting in termination of Gα- and Gβγ-mediated signaling to downstream effectors. RGS6 is a key regulator of γ-aminobutyric acid receptor signaling in the cerebellum. Mice lacking RGS6 exhibited abnormal gait and ataxia characterized by impaired rotarod performance [42]. RGS6 can also regulate parasympathetic activation in the heart and prevent parasympathetic override and severe bradycardia [22]. Loss of RGS6 predisposes the ventricle to prodeath signaling through a β_2_AR-GRK2-dependent signaling mechanism and plays a protective role in the ischemic heart [43]. RGS6 is also closely related to inflammatory diseases. For instance, spinal RGS6 expression was elevated upon spinal cord injury stimulation. RGS6 knockdown suppressed, while RGS6 overexpression aggravated, oxidative stress, inflammation and injury in mice [30]. RGS6 was also found to be upregulated in the livers of patients with nonalcoholic fatty liver disease. Human patients with high hepatic RGS6 expression exhibited a correspondingly high inflammatory burden, pronounced insulin resistance and poor liver function [29]. However, in this study, we found that RGS6 was downregulated under the high inflammatory burden in ALI (Fig. 2), in contrast to the results of previous studies. This inconsistency may be attributed to the different organs, etiologies, pathogeneses and molecular signaling pathways examined in these studies, and all of these potential reasons for the differing results need further study. Our study revealed that RGS6 deficiency increased the mortality of mice and led to more severe pathological damage in vivo upon LPS challenge (Fig. 3A–C). Moreover, compared to wild-type mice, RGS6 knockout mice exhibited more pronounced weight loss (Fig. 3D), increased neutrophil accumulation in lung tissue (Fig. 4A), an increased IL-6, IL-1β and MCP-1 level in BALF (Fig. 4E–G) and increased myeloperoxidase expression (Fig. 4C, D) after intratracheal instillation of LPS. The higher protein concentrations in BALF indicated greater impairment of the air‒blood barrier (Fig. 3E). RGS6 participates in regulating both G protein-dependent and -independent pathways. We speculated that RGS6 could target a kind of proinflammatory GPCR protein and restrict its expression during the pathological process of ALI/ARDS. Following RGS6 knocked out or downregulation, the related proinflammatory GPCR signaling was amplified, resulting in more pronounced pathological damage and more severe inflammation.

RGS6 has also been widely studied in oncology. It was recognized as a tumor suppressor and was shown to be downregulated in and protective against the development of various cancers. Decreased RGS6 expression was closely correlated with increased tumor size, TNM stage and lymphatic and distant metastasis [25]. Interestingly, some of the literatures reported low expression of RGS6 was associated with poor survival in lung cancer patients. RGS6 overexpression could suppress TGF-β-induced epithelial–mesenchymal transition and metastasis [28]. Some studies have also reported that RGS6 negatively regulates oncogene-induced cell transformation by targeting DNA methyltransferase 1, mediating the activation of ATM and p53 and promoting apoptosis [24, 26, 44]. In the present study, we found that RGS6 was able to decrease ROS production and inflammatory levels in macrophages (Fig. 7) and inhibit epithelial cell apoptosis (Fig. 8). In addition, we observed aggravated lung injury and more pronounced AEC2 loss upon RGS6 knockout (Fig. 5). RGS6 could influence the stemness, self-renewal and self-assembly capacity of AEC2s (Fig. 6). To distinguish the different roles of RGS6 in different disease models, it is crucial to precisely regulate RGS6 as well as clarify and improve the mechanism research. G protein-dependent and -independent pathways should all be considered and verified in future mechanistic studies.

GPCRs are important targets for drug development and are involved in many popular topics in therapeutic research. RGS family proteins are also attracting increasing attention in drug development due to their close relationship with GPCRs. In regard to mature products related to RGS family proteins, the most research to date has focused on RGS4 as a drug. For instance, intrathecally administration of CCG50014, an RGS4 inhibitor, attenuated nociceptive responses and enhanced opioid-mediated analgesic effects in the mouse formalin test due to the role of RGS4 in inactivating GPCRs related to opioid receptors [45]. Downregulation of RGS4 by CCG63802, another selective, reversible and allosteric RGS4 inhibitor, significantly increased inflammatory cell accumulation, airway hyper-responsiveness and IL-4 level in BALF [46]. To date, few studies have been focused on RGS6 and the respiratory system. We hope that our study will provide some insights useful for the development of drugs to treat ALI/ARDS. RGS6 may be a target for drug development in this context.

**Fig. 9:**
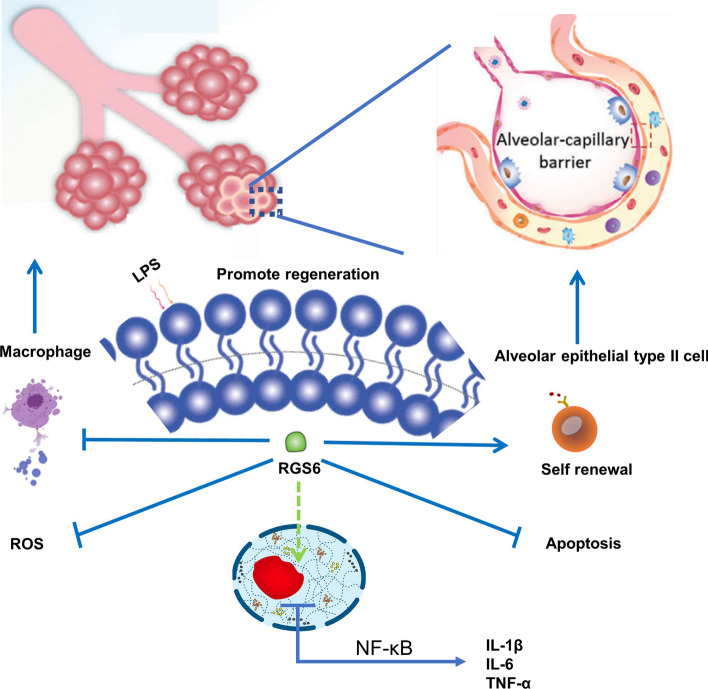
Schematic diagram showed the key roles of RGS6 in acute lung injury

In this study, we found that RGS6 protects against ALI via two mechanisms. On one hand, RGS6 overexpression reduced ROS production and proinflammatory cytokine transcription in macrophages. On the other hand, RGS6 inhibited epithelial cell apoptosis and promoted the stemness and self-renewal capacity of AEC2s (Fig. 9). Moreover, RGS6 deficiency aggravated lung injury and increased mortality in mice. Therefore, RGS6 may be a potent clinical therapeutic target to improve the outcomes of ALI/ARDS patients. This is the first study to show the role of RGS6 in ALI/ARDS. Thus, the literatures to which we can refer are limited. More detailed mechanistic studies will be completed in the future.

Our study has some limitations. For instance, our results were not validated in clinical specimens due to the difficulty of obtaining lung tissue samples from ALI/ARDS patients. Moreover, the specific mechanisms of the role of RGS6 in ALI/ARDS need further elucidation.

## Conclusions

RGS6 plays a protective role in acute lung injury not only in early inflammatory responses but also in endogenous lung stem cell regeneration. RGS6 may provide a new therapeutic target for the discovery of drugs to improve curative effect of ARDS treatment.

### Supplementary Information

**Additional file 1: **Supplementary materials.

## Data Availability

All data generated or analyzed during this study are included in this published article. The datasets used and/or analyzed during the current study are available from the corresponding author on reasonable request.

## Abbreviations

ARDS: Acute respiratory distress syndrome
ALI: Acute lung injury
AEC2s: Alveolar epithelial type II cells
AEC1s: Alveolar epithelial type I cells
ATM: Ataxia Telangiectasia Mutated protein
BALF: Bronchoalveolar lavage fluid
BP: Biological process
CC: Cellular component
CRGs: Congruously regulated genes
DEGs: Differentially expressed genes
ELISA: Enzyme-linked immunosorbent assay
GPCR: G protein-coupled receptors
GTP: Guanosine triphosphate
GO: Gene Ontology
HE: Hematoxylin–eosin
IL-6: Interleukin 6
IL-1β: Interleukin-1 Beta
Ly6g: Lymphocyte antigen 6 family member G
iNOS: Nitric Oxide Synthase, Inducible
KEGG: Kyoto Encyclopedia of Genes and Genomes
LPS: Lipopolysaccharide
MPO: Myeloperoxidase
MCP-1: Monocyte Chemotactic Protein 1
MF: Molecular function
PBS: Phosphate-buffered saline
PA: Pseudomonas aeruginosa
PDPN: Podoplanin
qRT-PCR: Quantitative Real-time Polymerase Chain Reaction
RGS6: Regulator of G protein signaling protein 6
ROS: Reactive oxygen species
SFTPC: Surfactant Protein C
SCGB1A1: Secretoglobin Family 1A Member 1
TNF-α: Tumor Necrosis Factor-Alpha
TNM: Tumor, Lymph Node, Metastasis
TUNEL: Terminal deoxynucleotidyl transferase dUTP nick end labeling
WT: Wild type
