# Supplementary material for: Regulator of G protein signaling protein 6 alleviates acute lung injury by inhibiting inflammation and promoting cell self-renewal in mice
Source: Cell Mol Biol Lett. 2023 Dec 8;28:102. doi: 10.1186/s11658-023-00488-z (PMC10709870; doi:10.1186/s11658-023-00488-z)

**Supplementary Materials**

**Supplementary Figure 1.** **Phenotypes comparison between WT and RGS6^-/-^ mice without LPS administration *in vivo*.** A. HE staining showed pulmonary histological structures in different groups. Scale bar: upper 200 μm, lower 100 μm. B. Protein concentration in BALF in different groups. C. IL-6 concentration in BALF in different groups. D. IL-1β concentration in BALF in different groups. E. MCP-1 concentration in BALF in different groups. F. Immunohistochemistry showed neutrophils in lung tissues in different groups. Neutrophils were stained by anti-Ly6g antibody. Scale bar: 50 μm. WT, wild type mice group; RGS6^-/-^, RGS6^-/-^ mice group. Data are shown as mean± SEM. ^**^ *P*<0.01 vs. WT group; ^ns^ no significance vs. WT group.


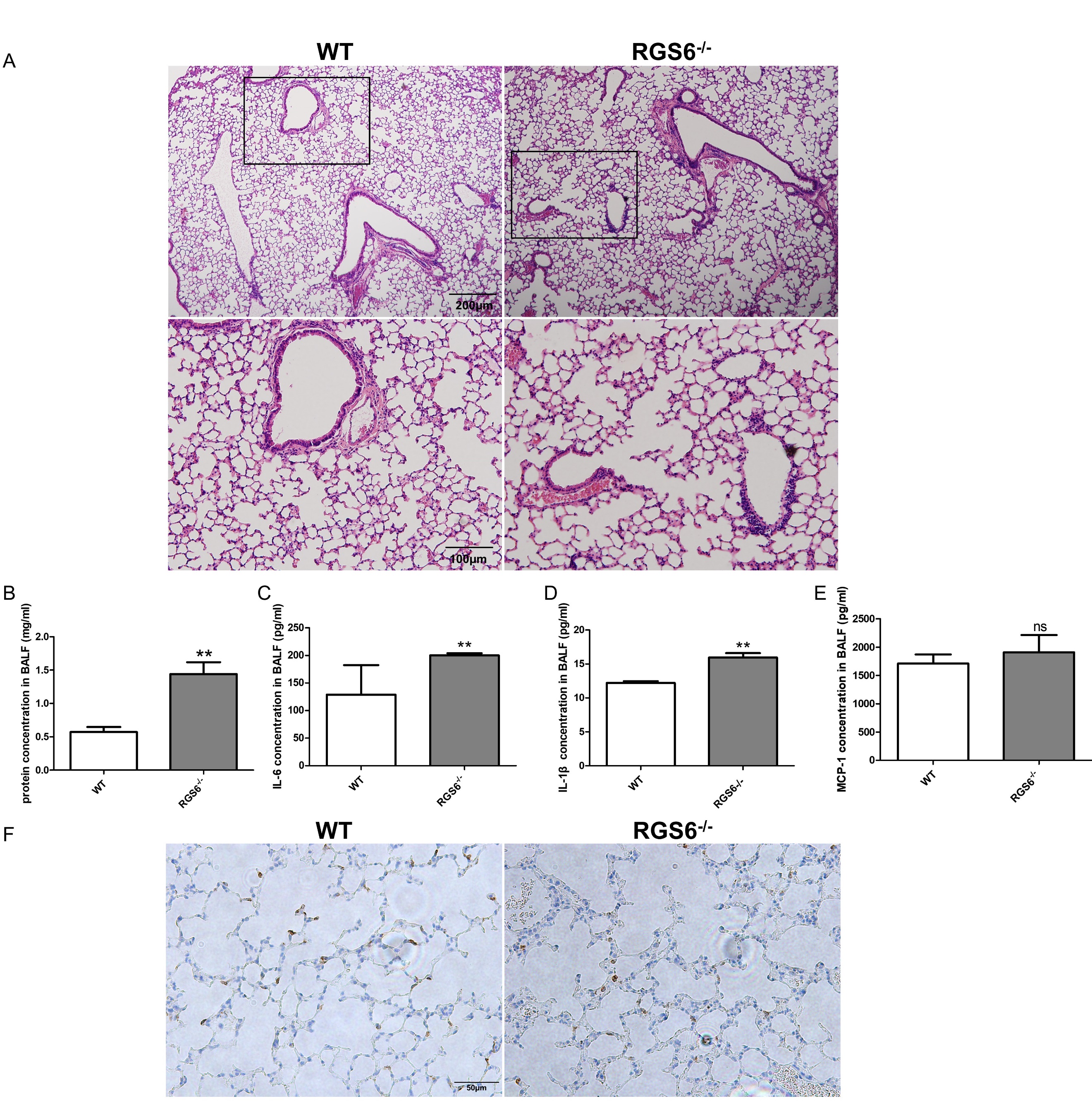


**Supplementary Figure 2. Inhibition control of Caspase 3 activity assay.** MLE-12 cells was pretreated by Caspase 3/7 inhibitor Ac-DEVD-CHO and then added with substrate for Caspase 3 activity detection based on SuperView 488 fluorescence signal. A. Cell nucleus was stained by Hoechst 33342. B. Caspase 3 activity was indicated by SuperView 488. C. The merged image.


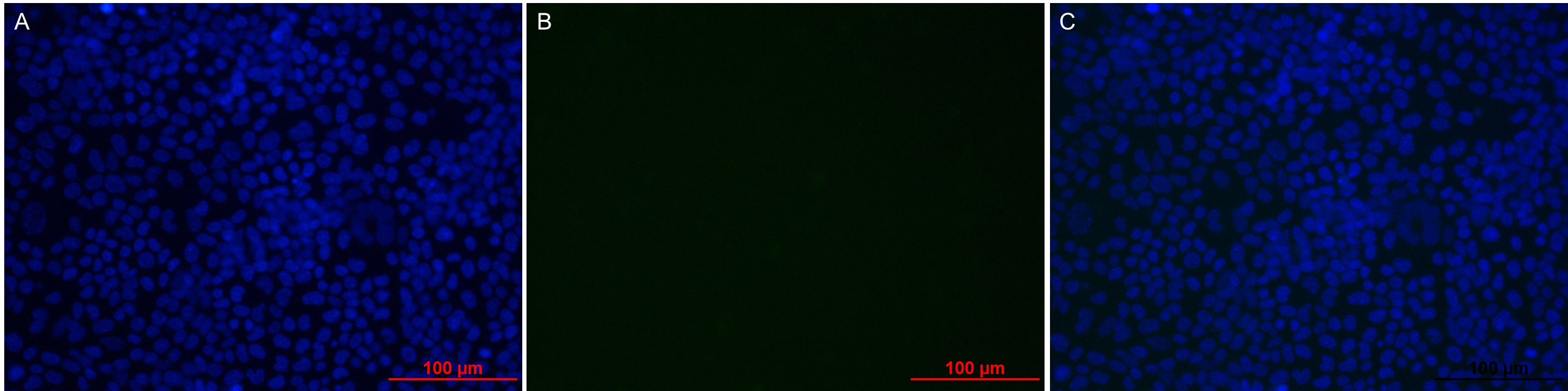


**Uncropped images of blots shown throughout the manuscript.**

**Figure 2C**

GAPDH


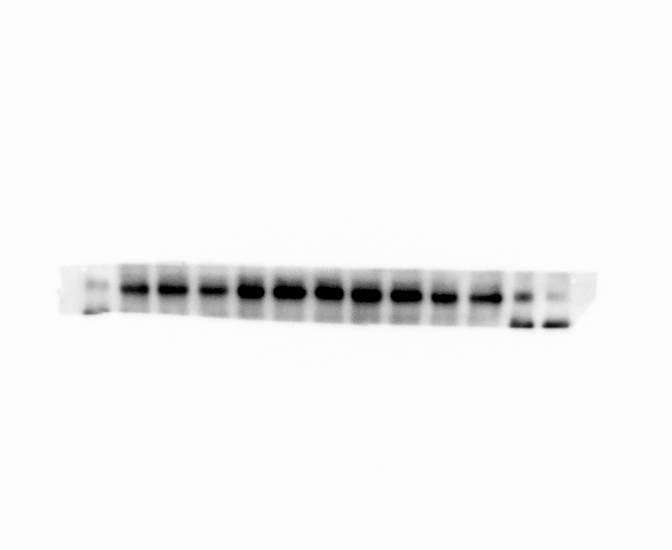


LPS

PBS

RGS6


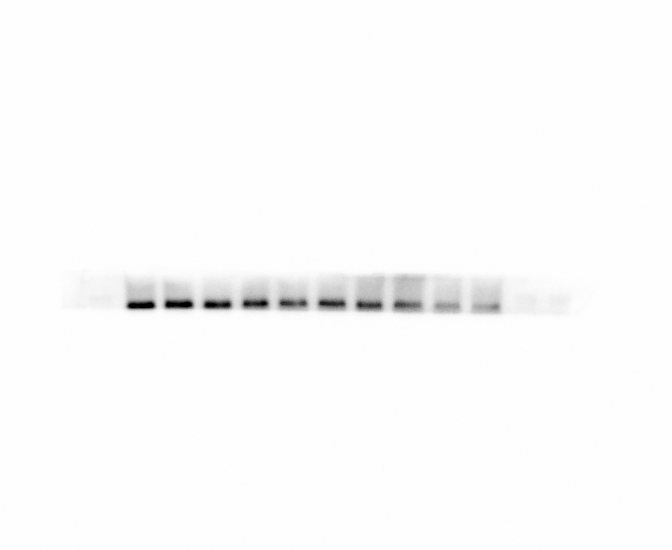


LPS

PBS

**Figure 2F**

**GAPDH**

C LPS C LPS


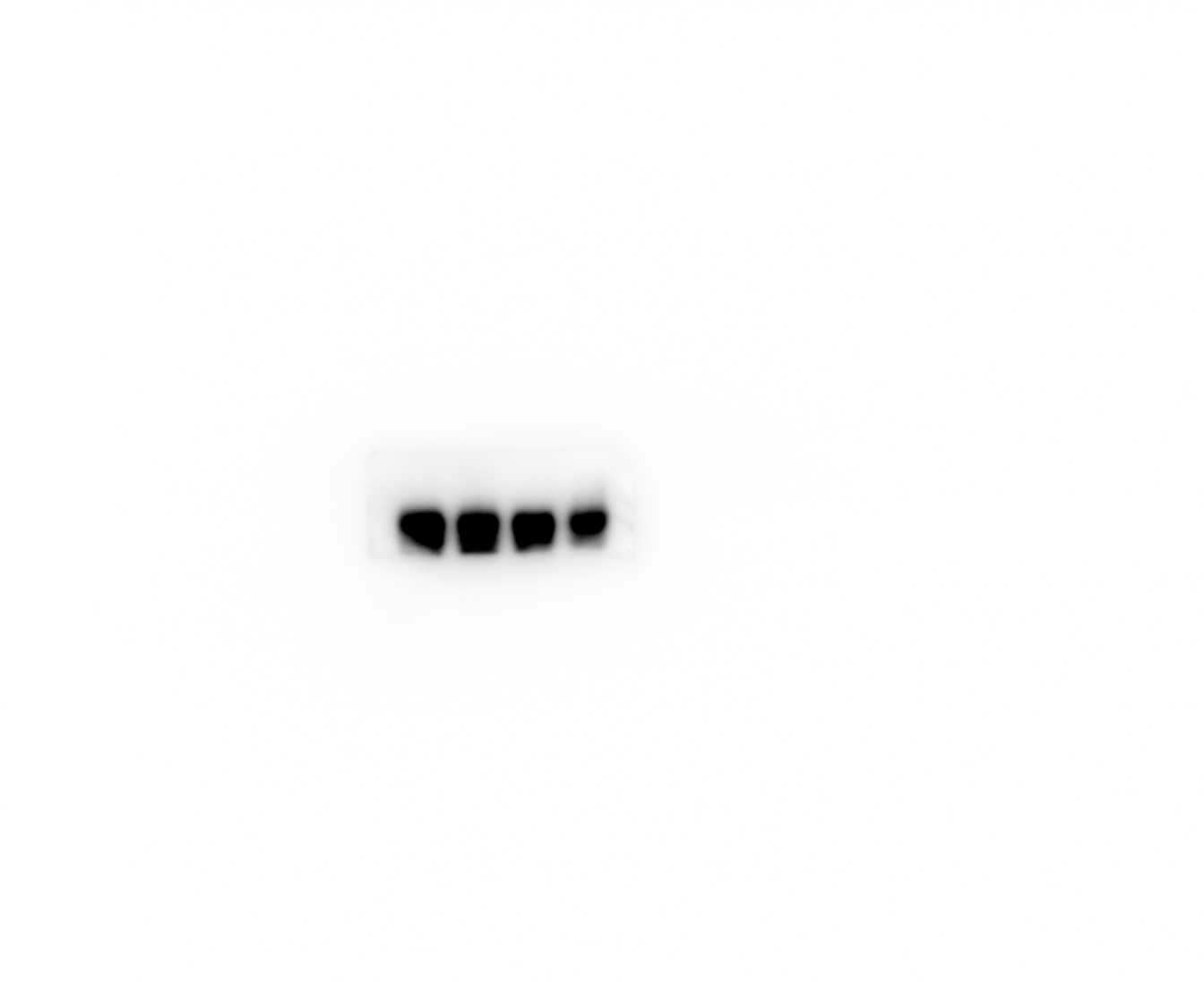


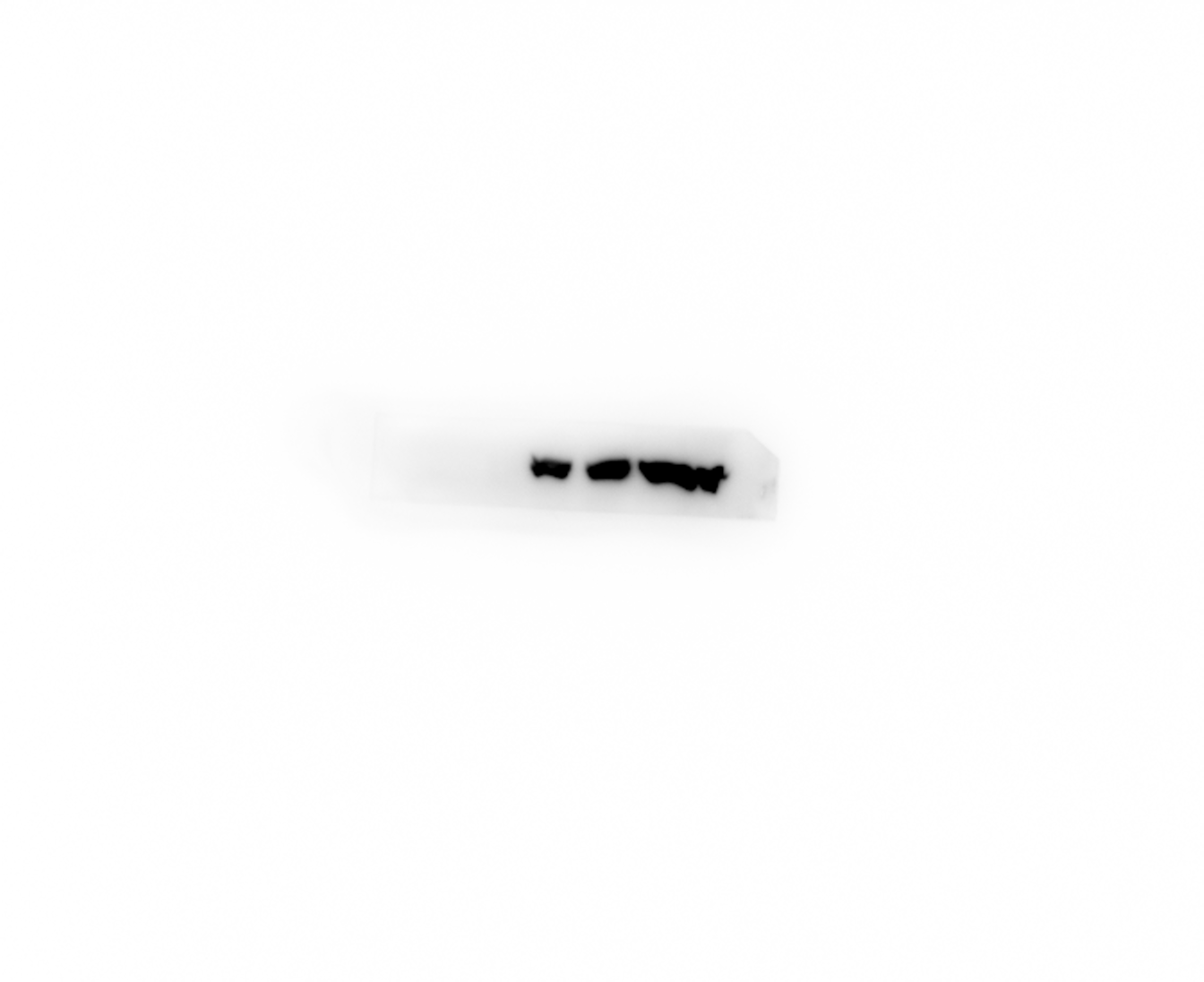


C LPS C LPS

C LPS

**RGS6**

C LPS C LPS


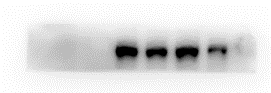

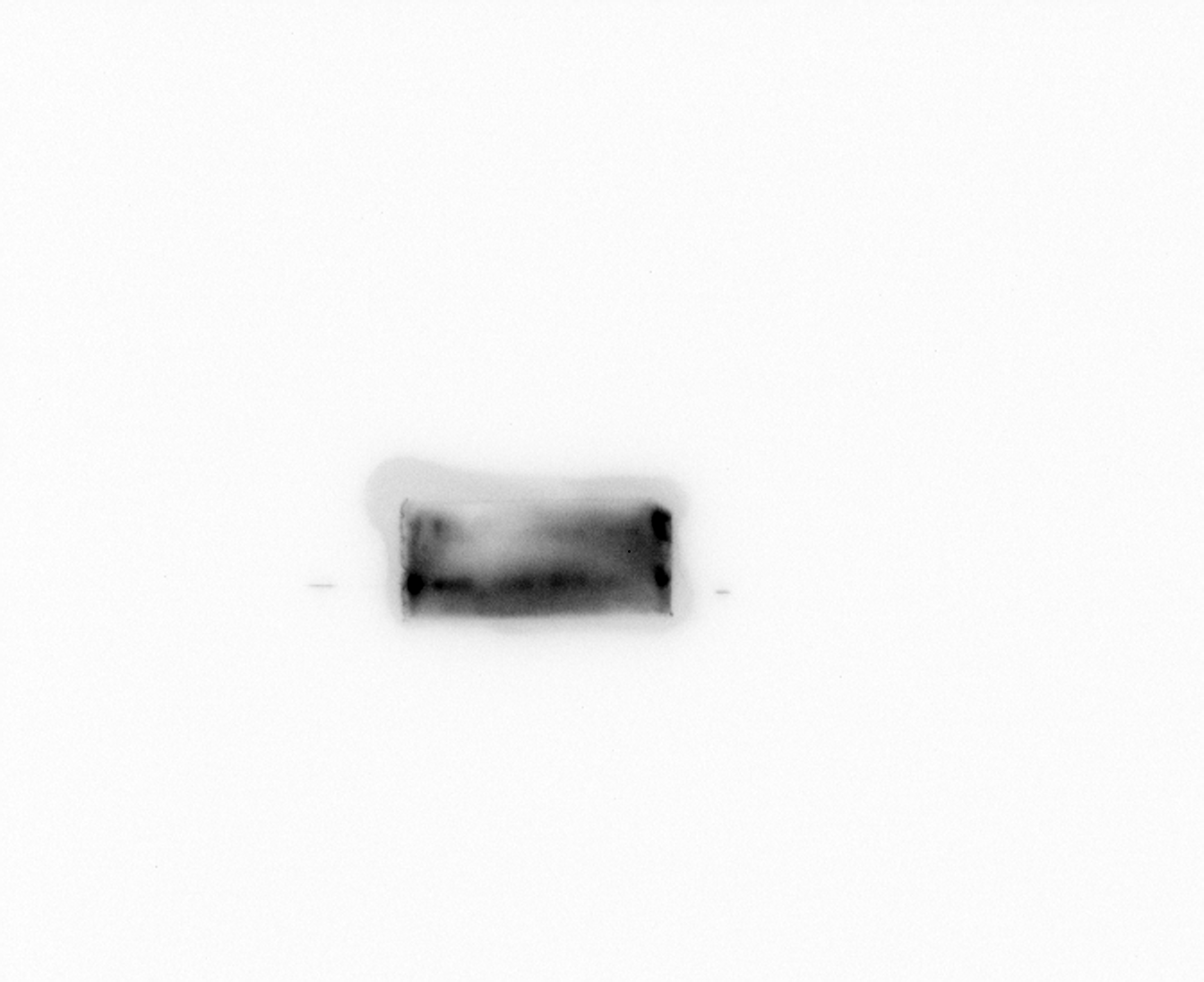


C LPS C LPS

**Figure 2H**

**GAPDH**

C LPS H_2_O_2_

**
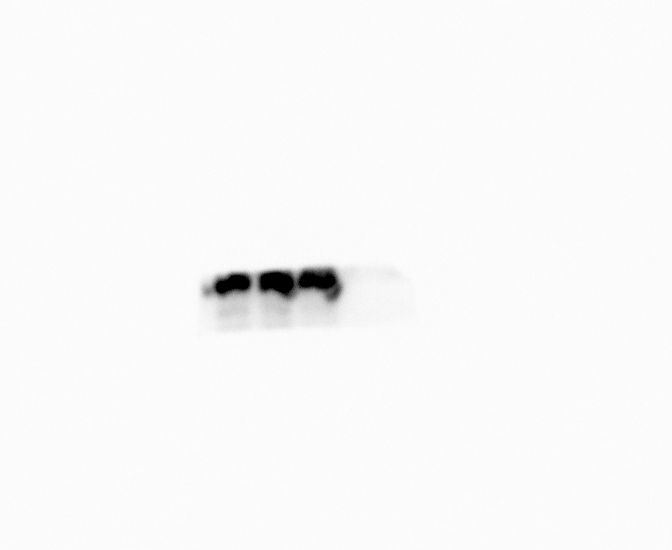
**

**β-actin**

C LPS C LPS


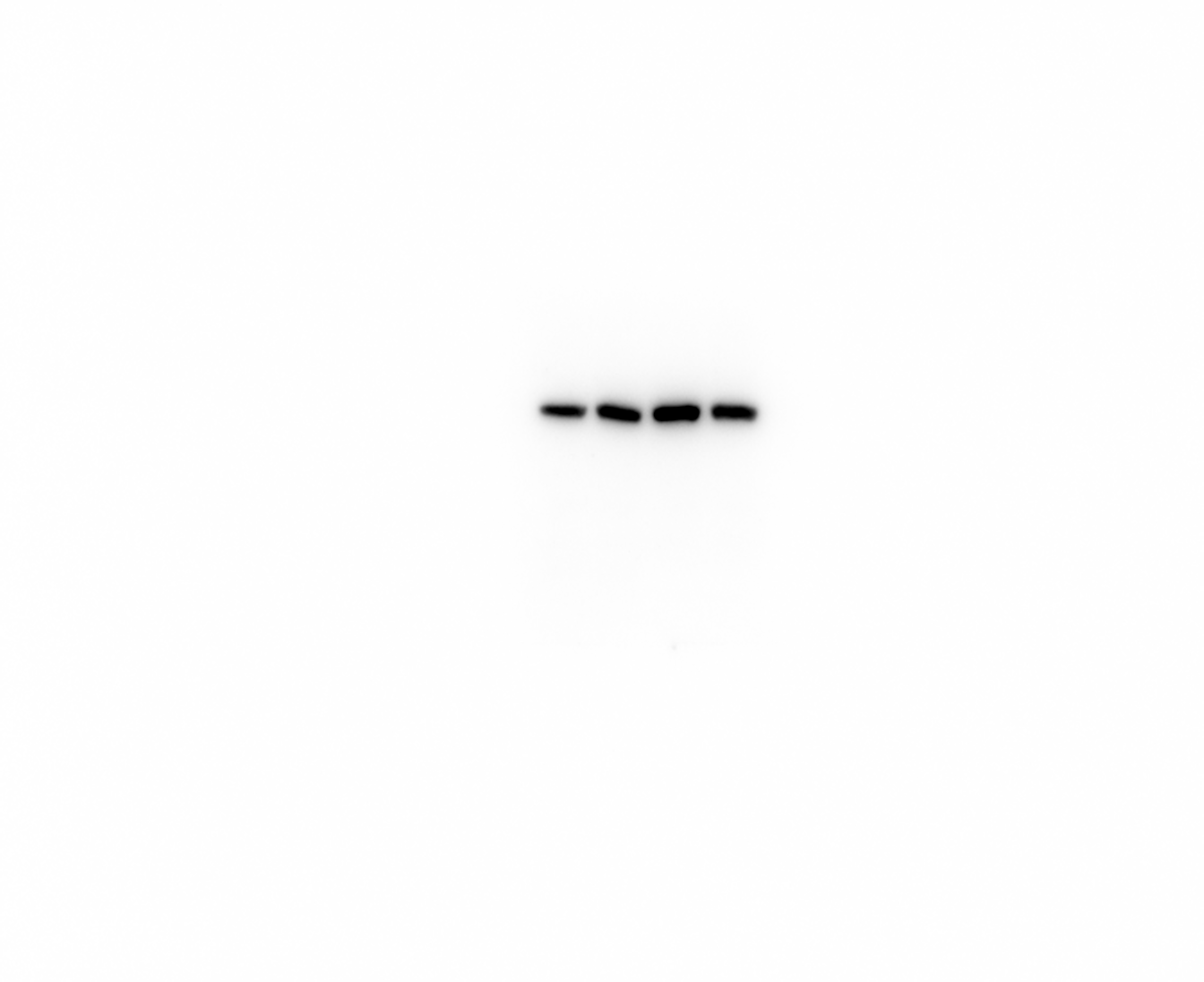


**RGS6**

C LPS H_2_O_2_


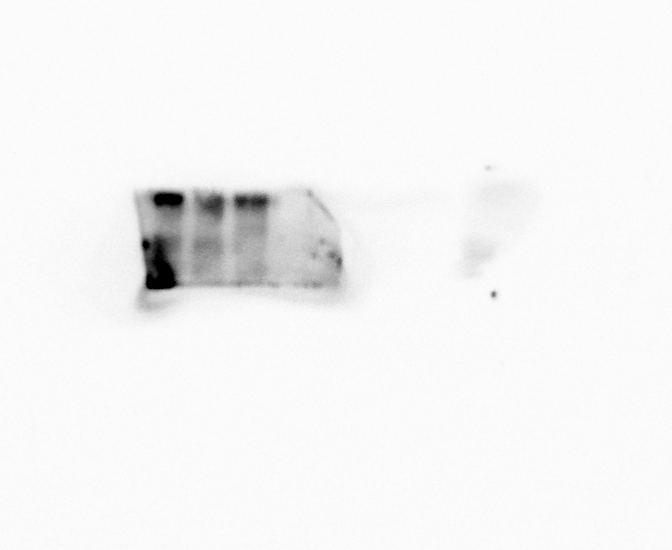


C LPS C LPS


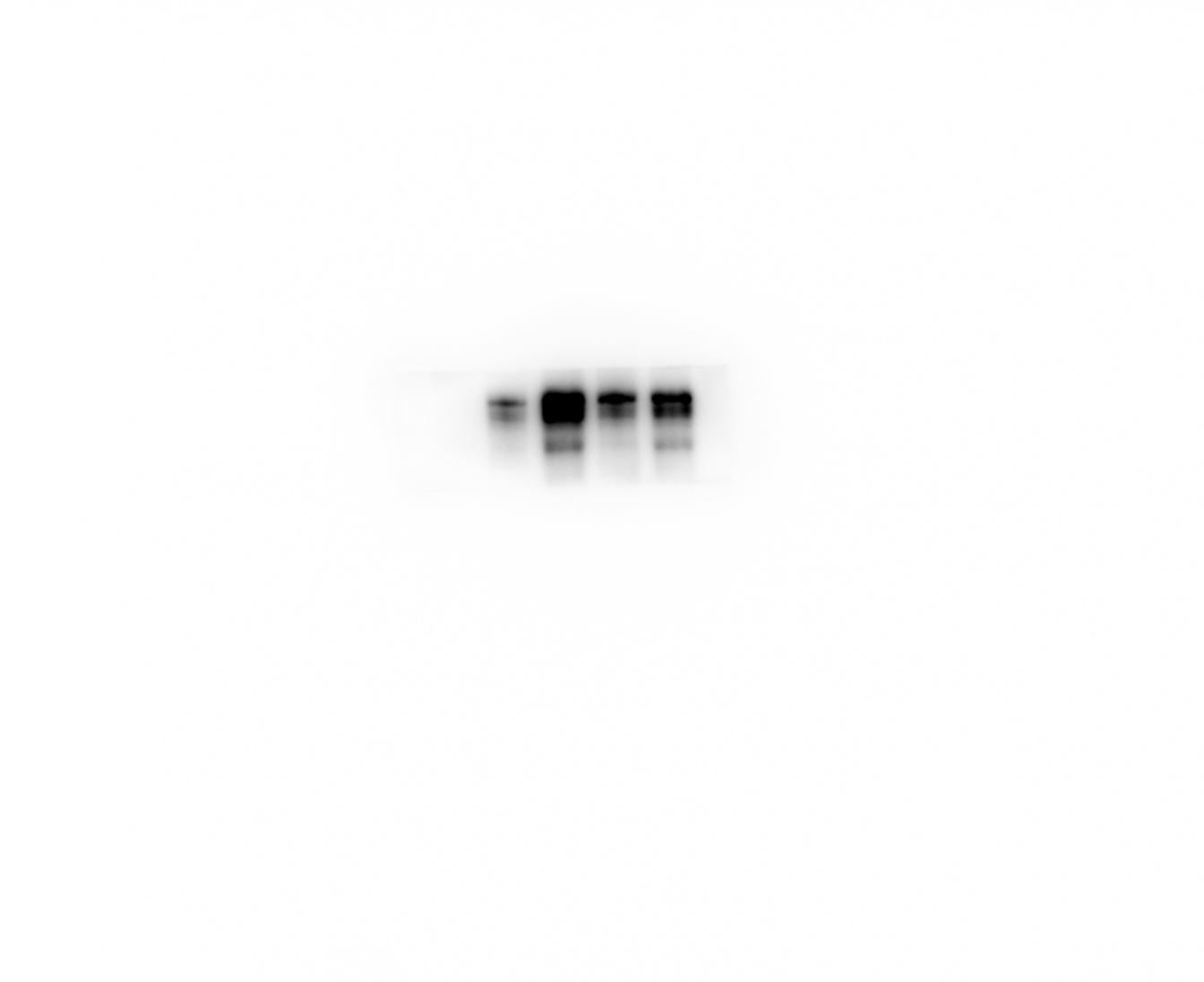


**Figure 4C**

**β-actin**


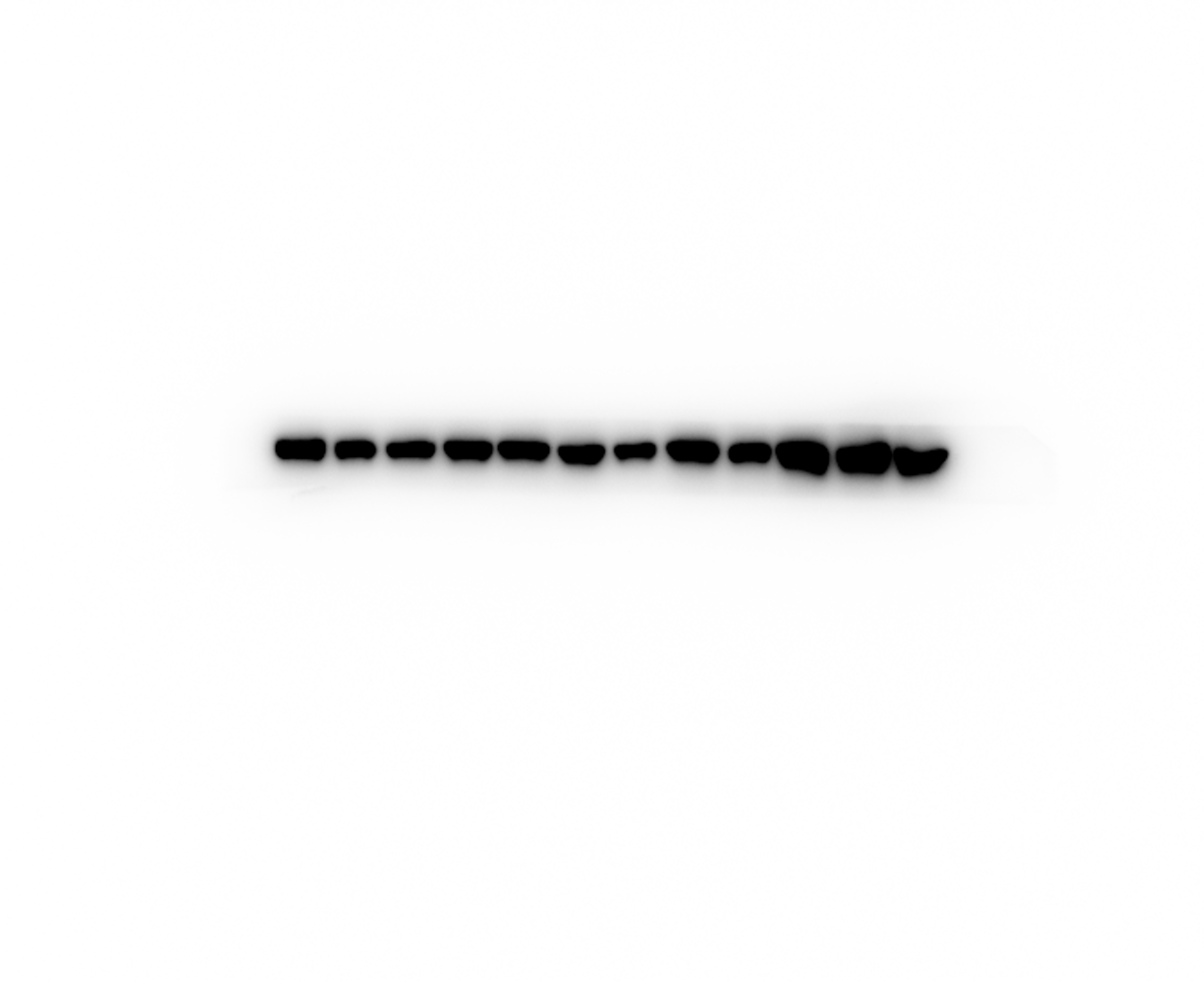


RGS6^-/-^ LPS

WT LPS

WT PBS

**MPO**


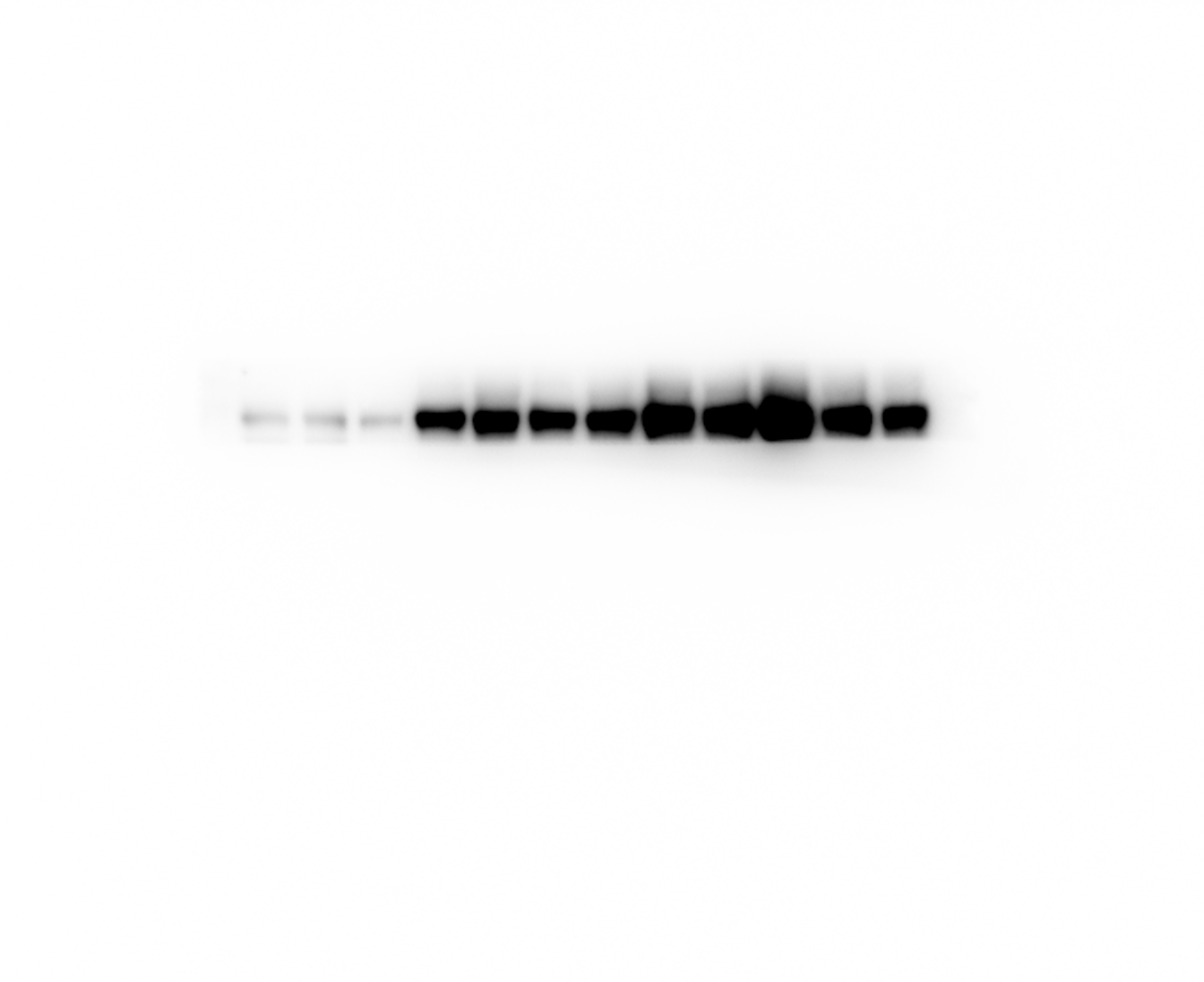


WT PBS

RGS6^-/-^ LPS

WT LPS

**Figure 7C**

**β-actin**


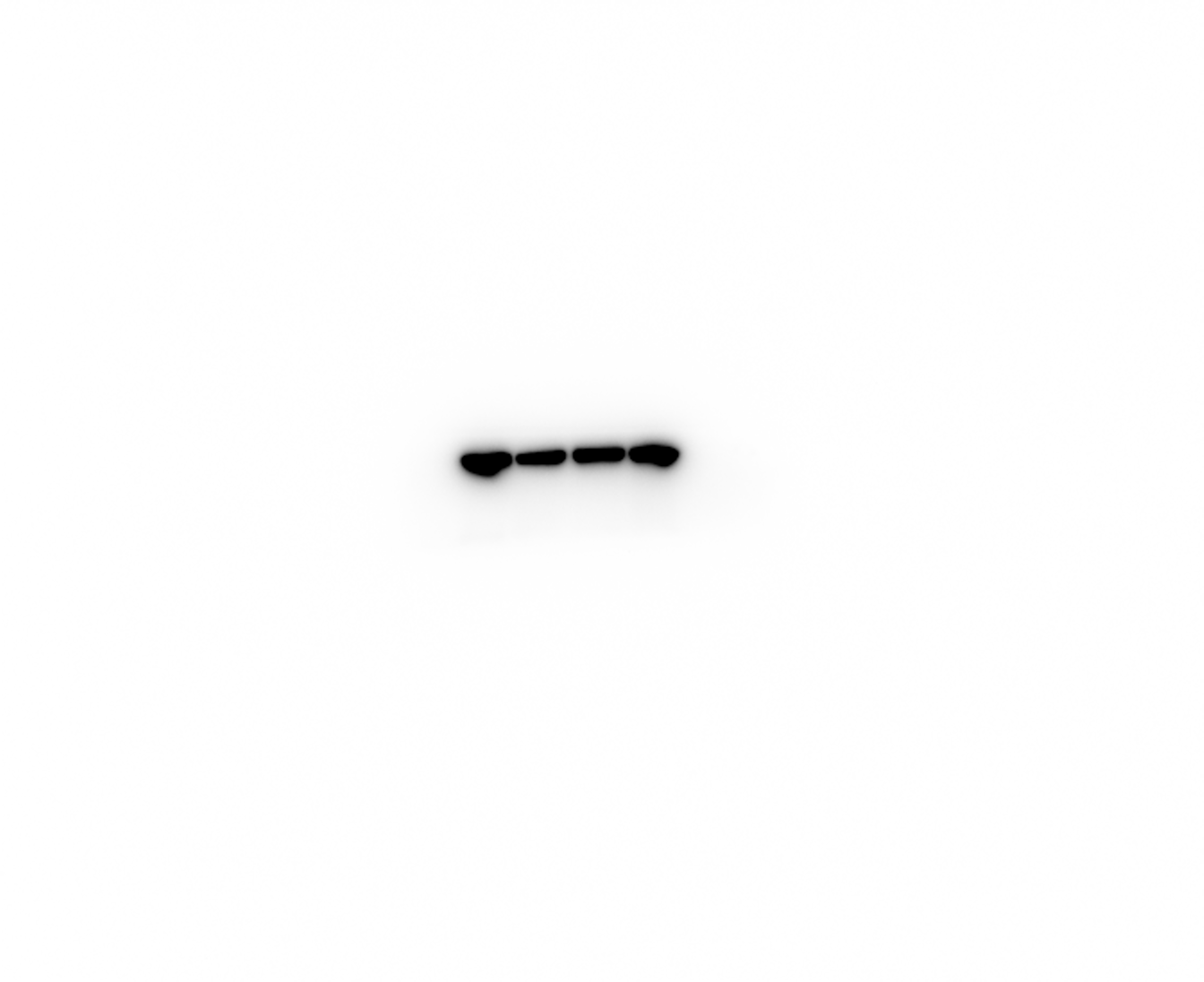


NC OE NC OE

**GAPDH**

NC OE NC OE


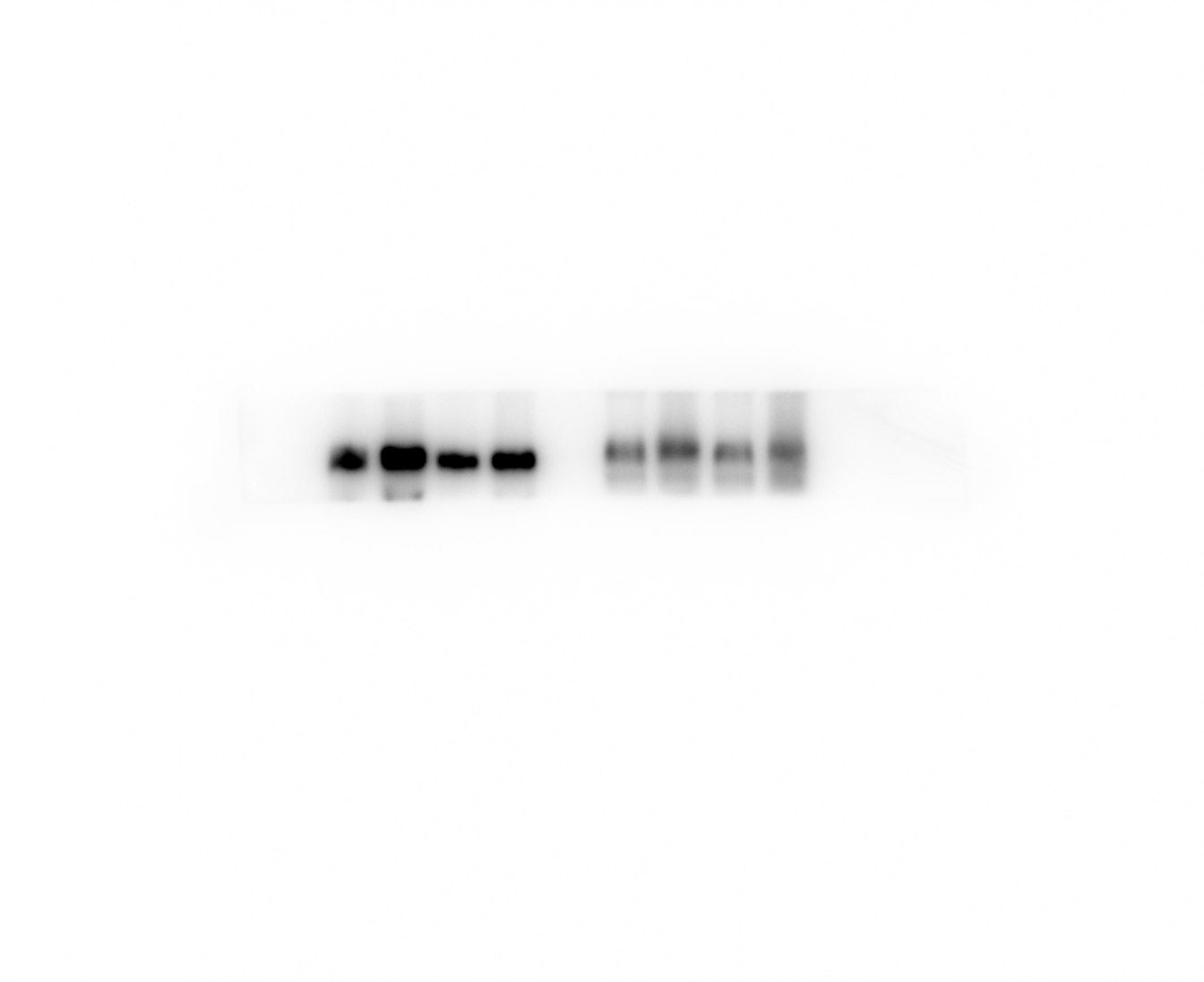


**
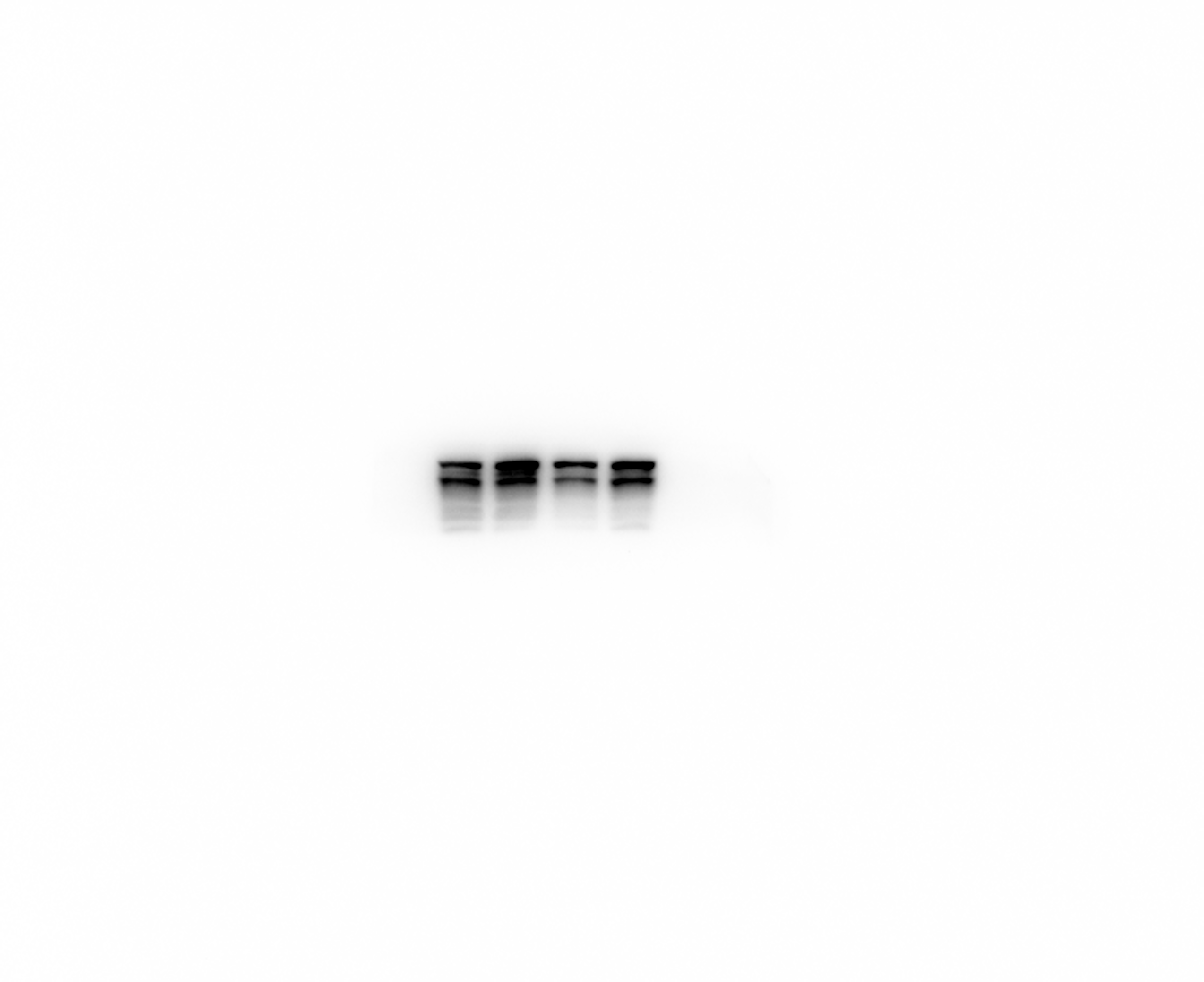

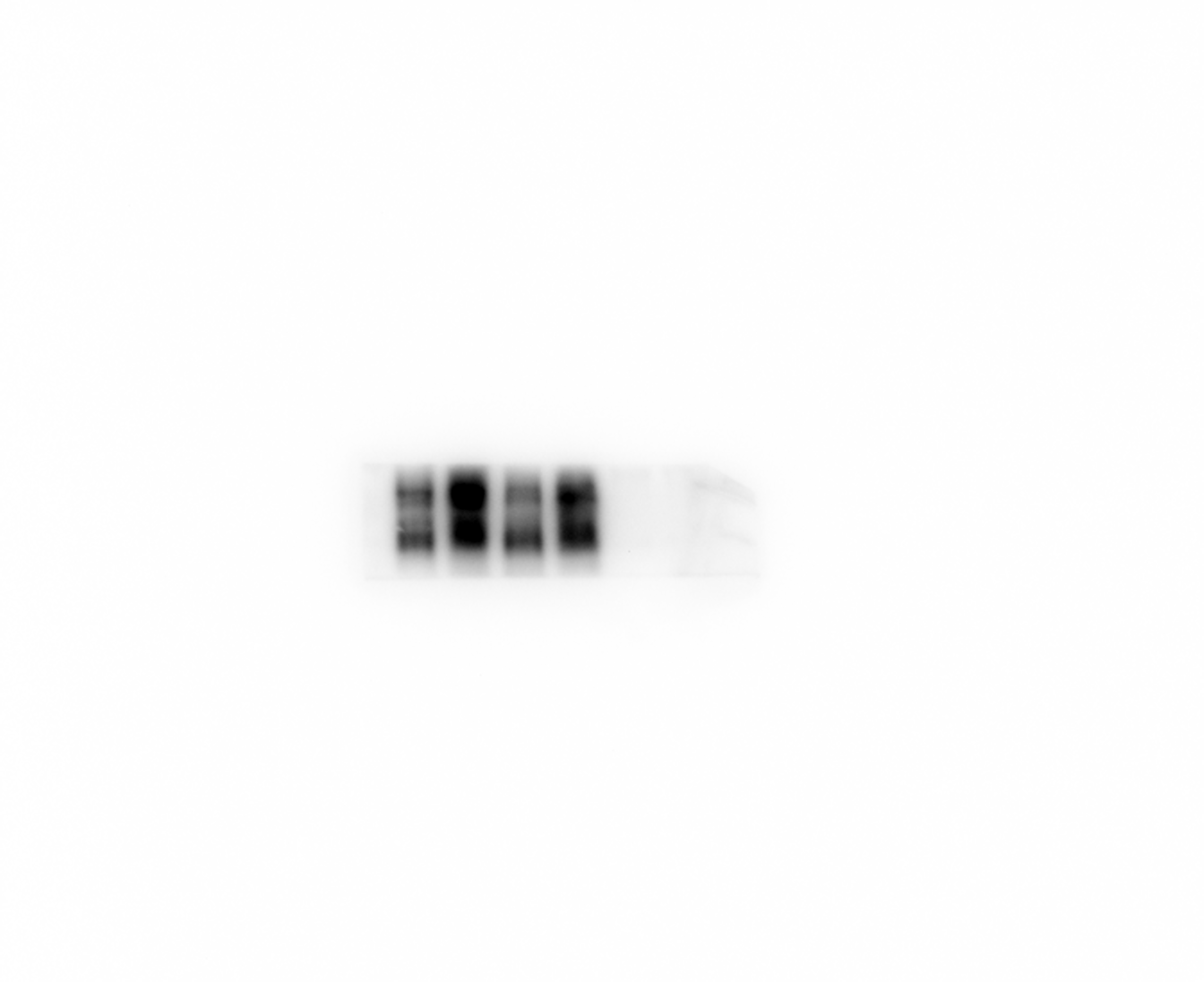
RGS6**

NC OE NC OE

NC OE NC OE

**Figure 8F**

**β-actin
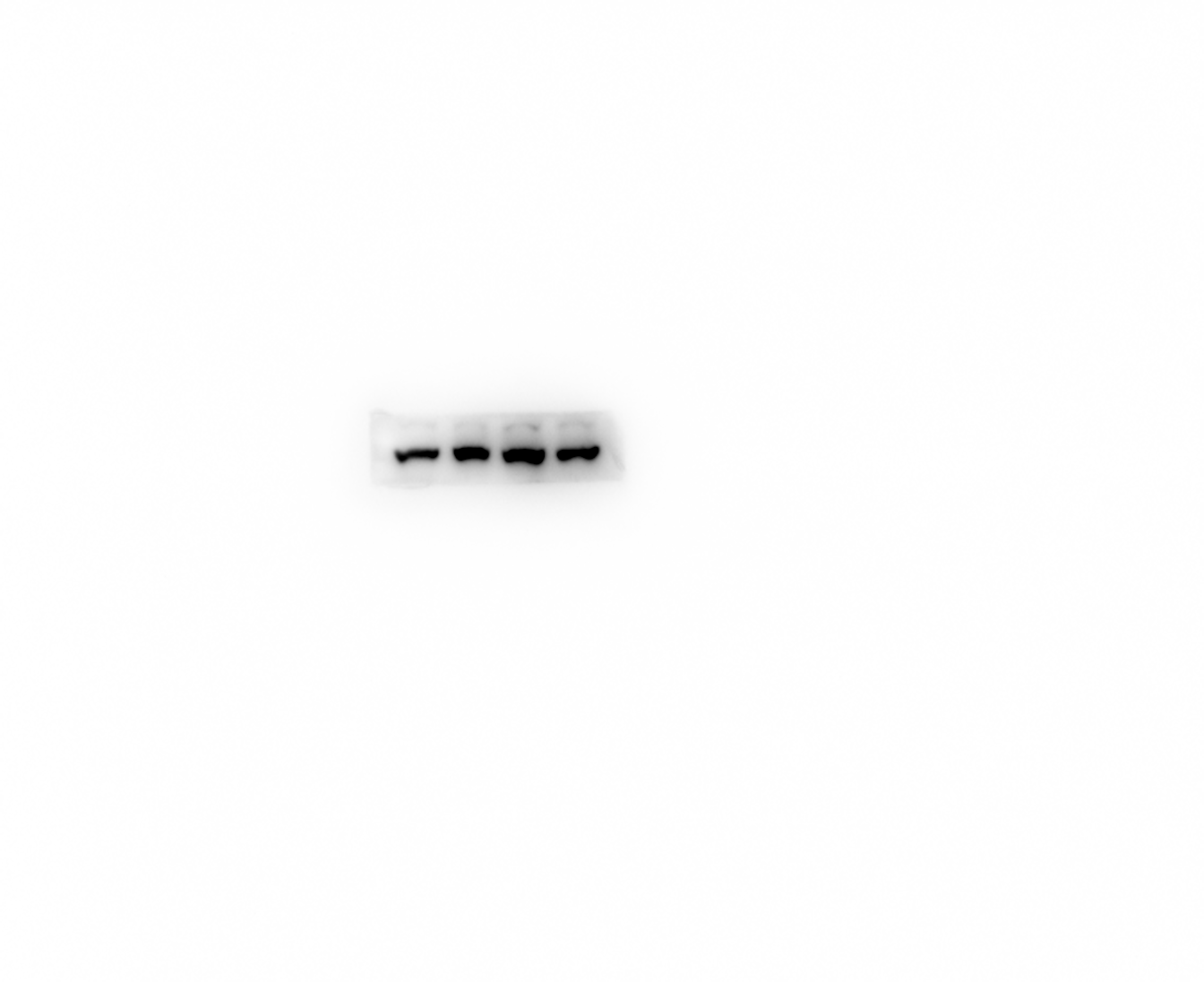
**

NC-C OE-C NC-LPS OE-LPS


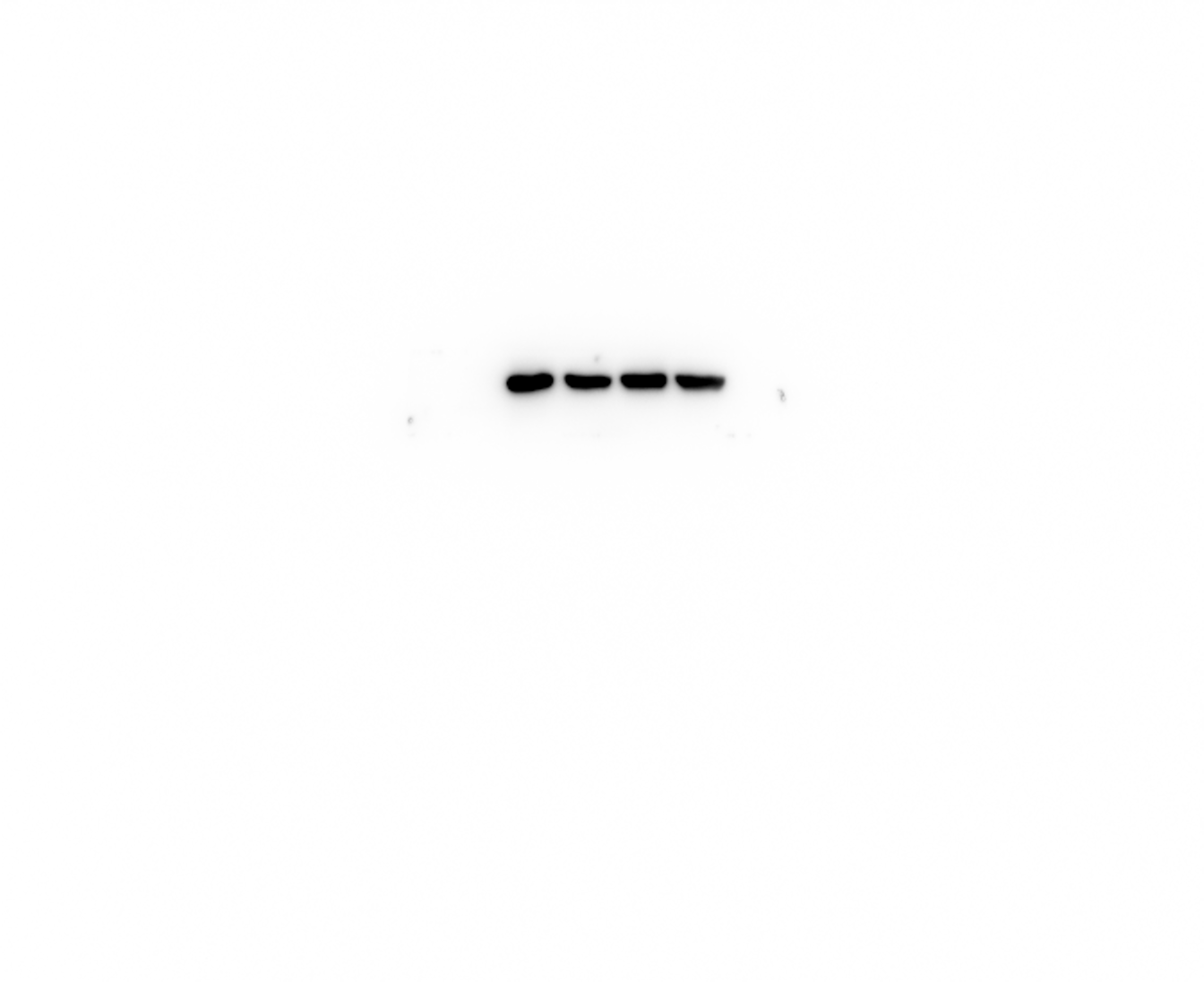


NC-C OE-C NC-LPS OE-LPS

NC-C OE-C NC-LPS OE-LPS


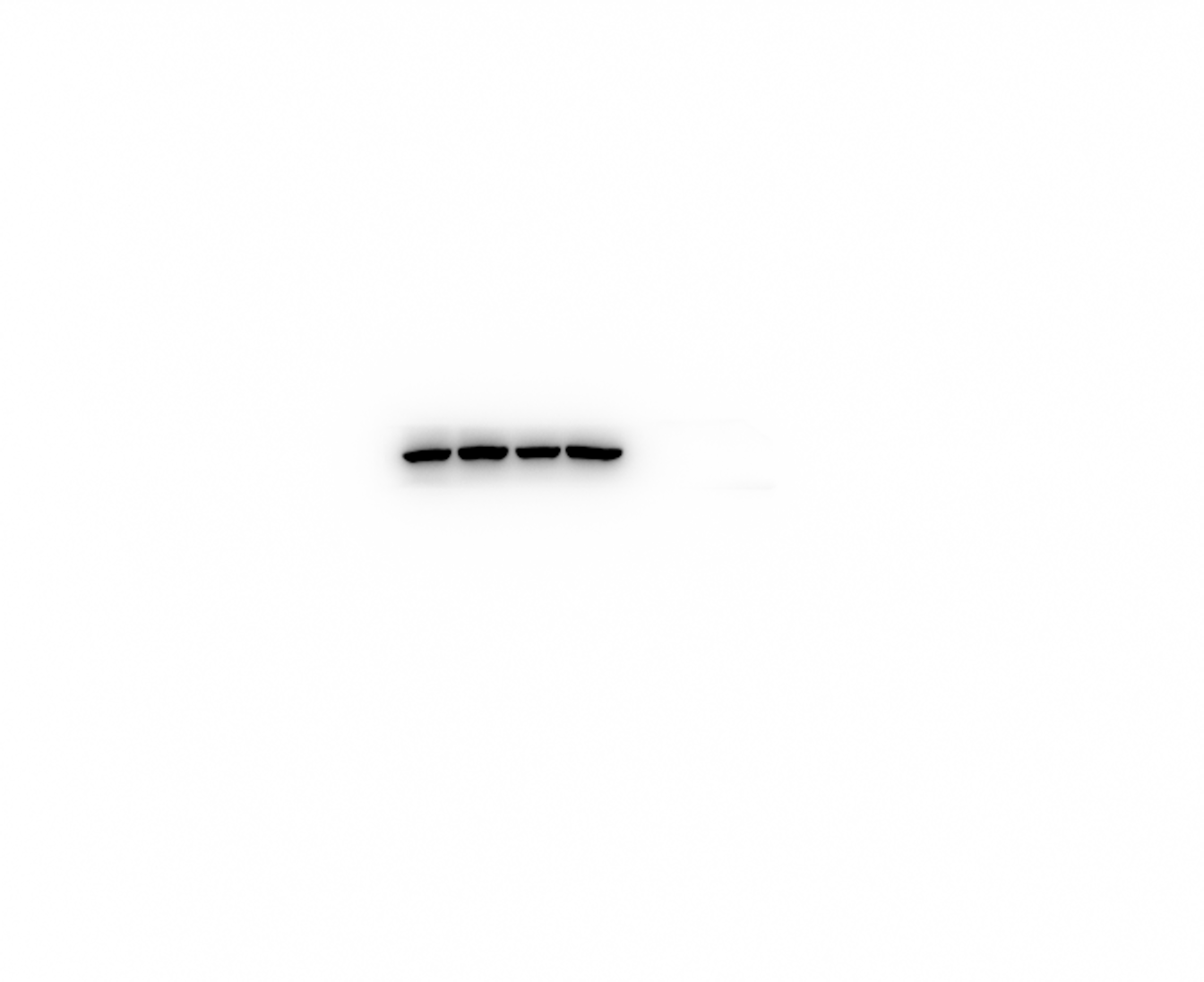


**p-p65**

NC-C OE-C NC-LPS OE-LPS


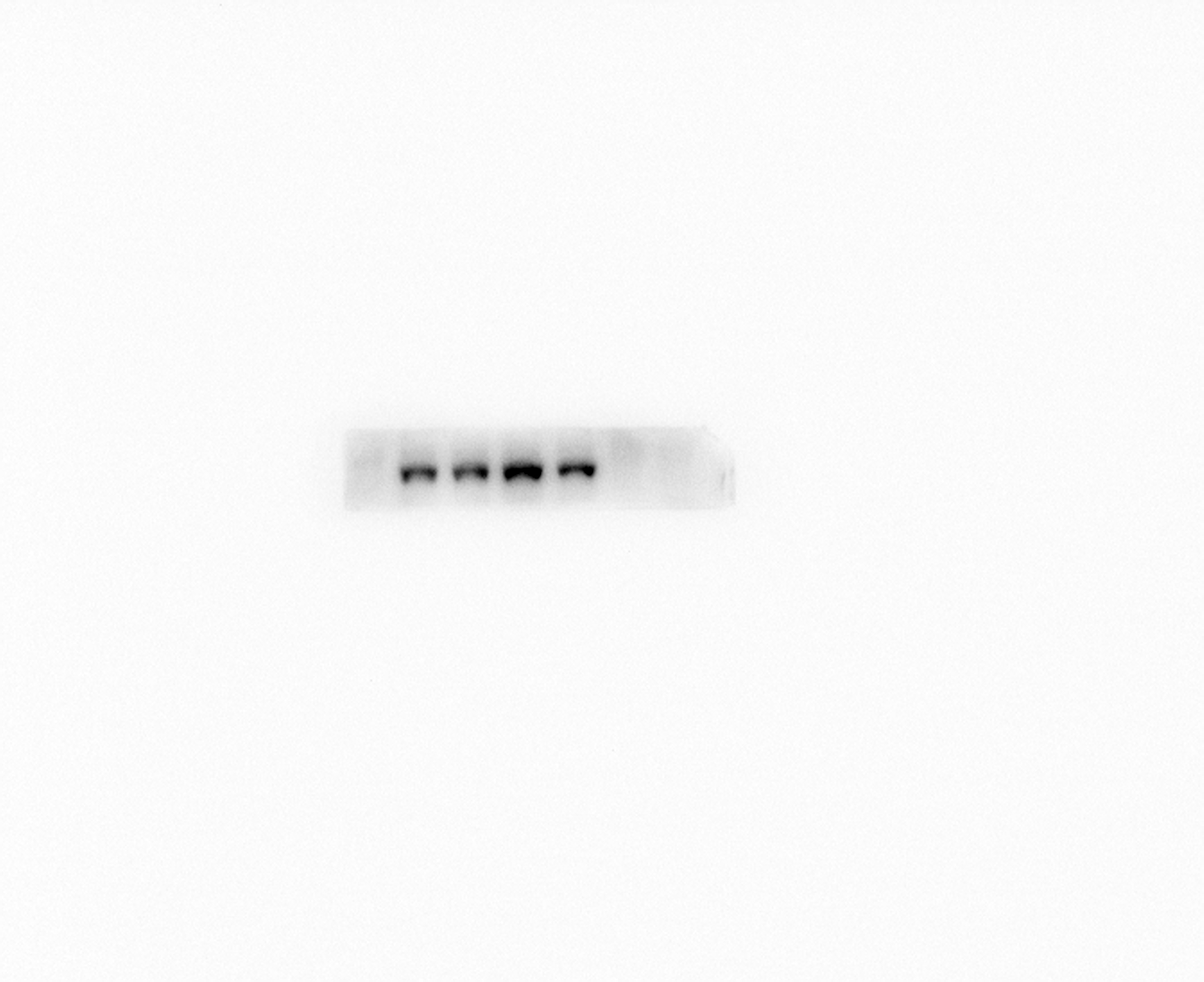


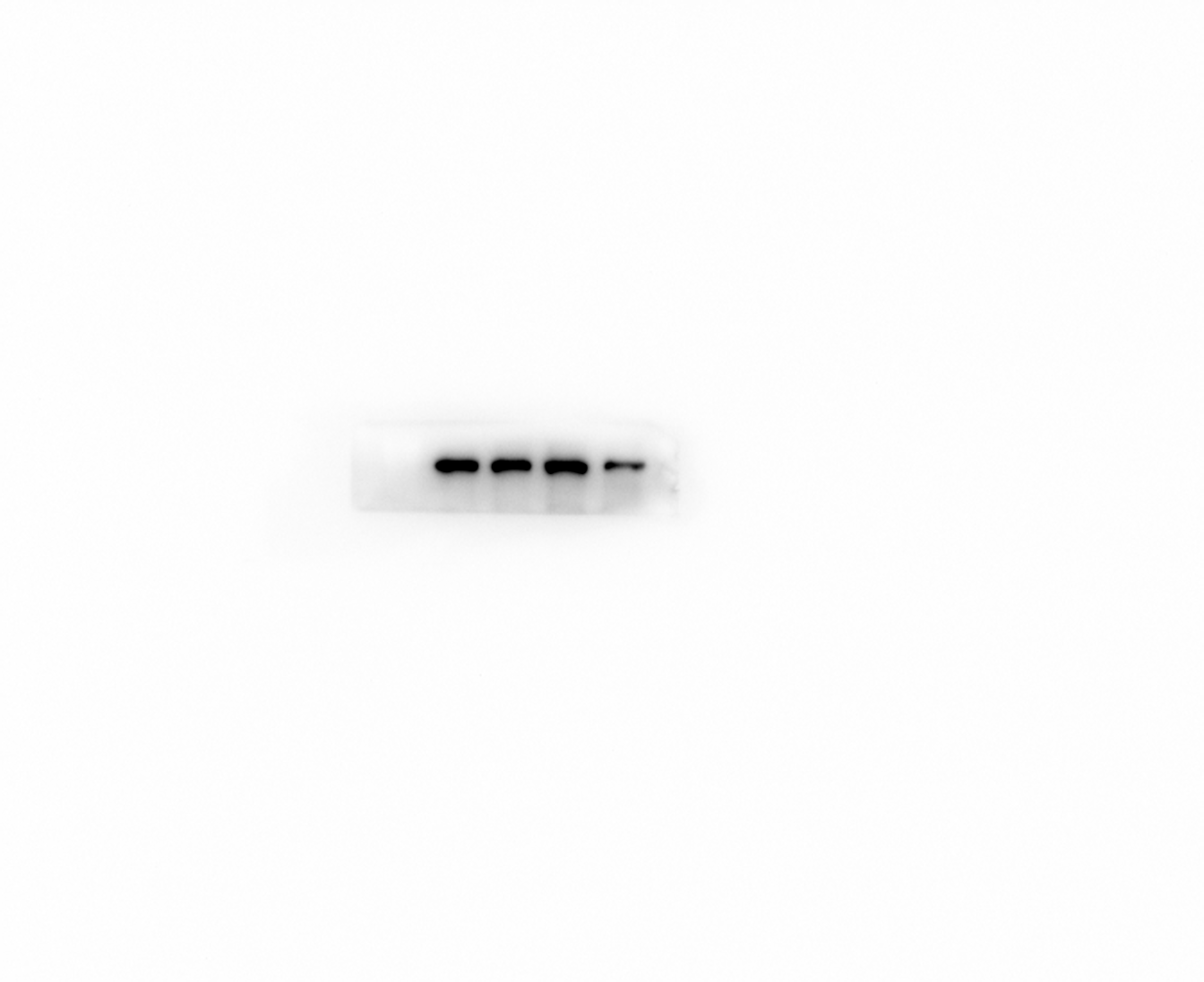


NC-C OE-C NC-LPS OE-LPS

NC-C OE-C NC-LPS OE-LPS


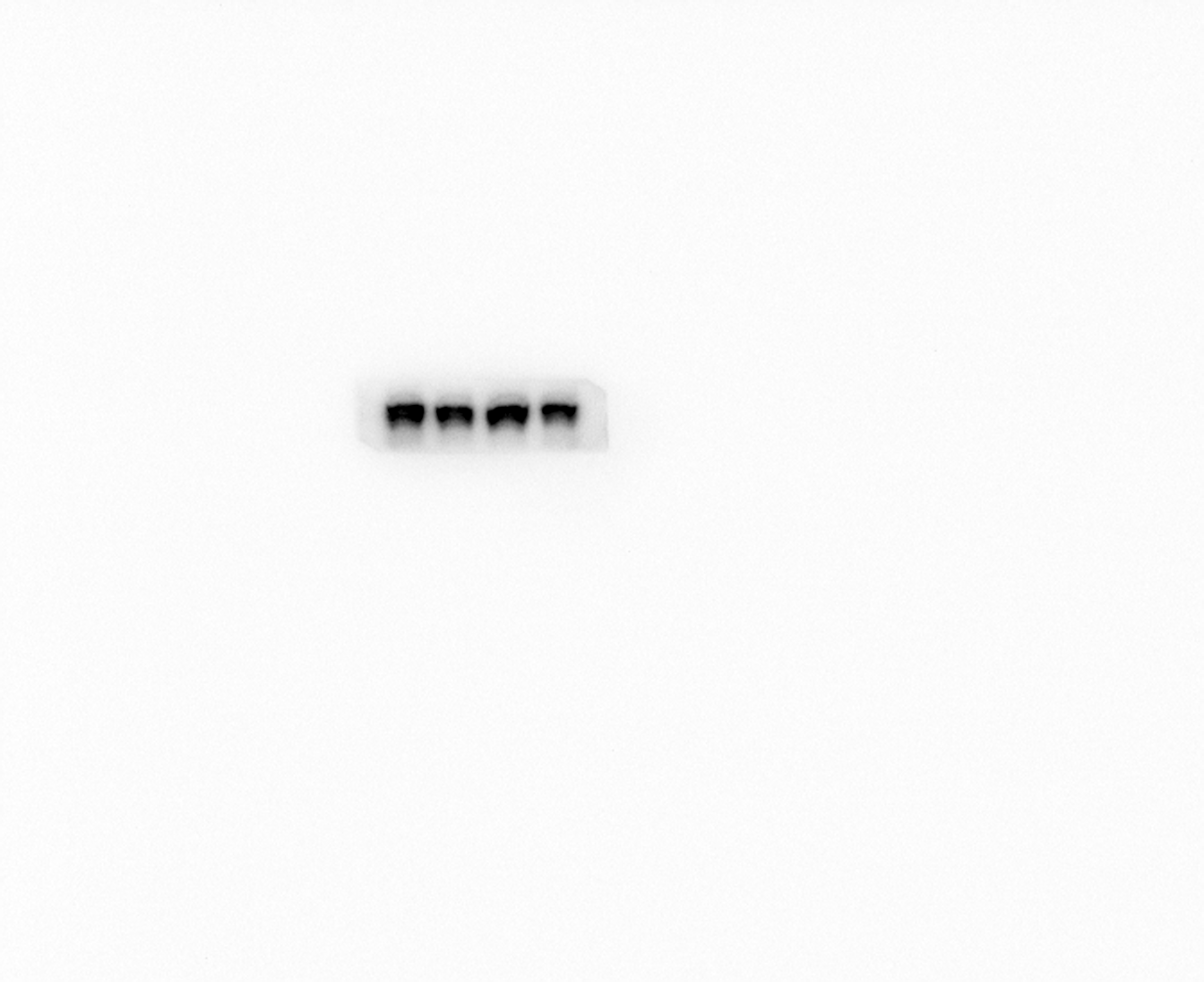


**Bax**

NC-C OE-C NC-LPS OE-LPS


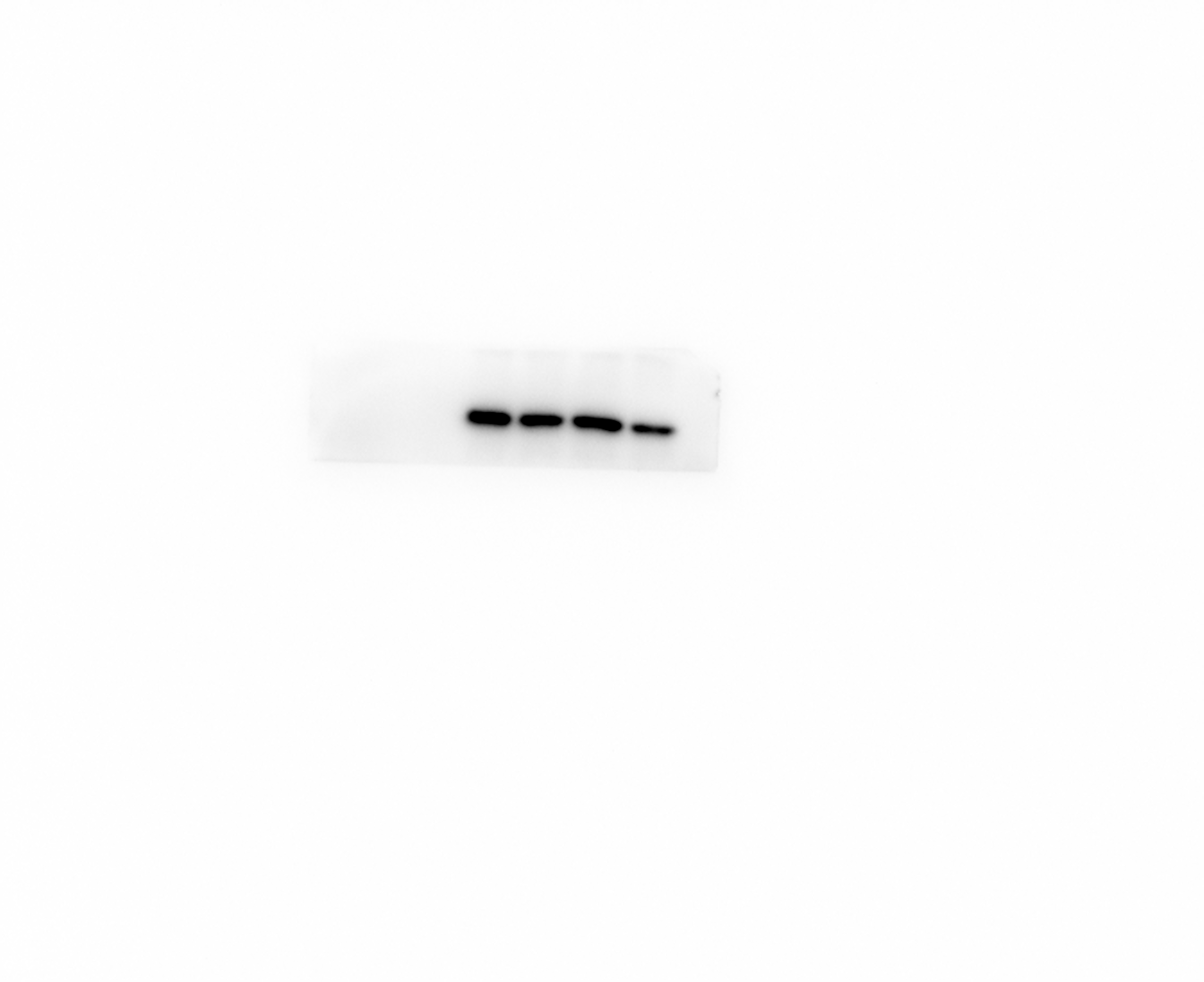


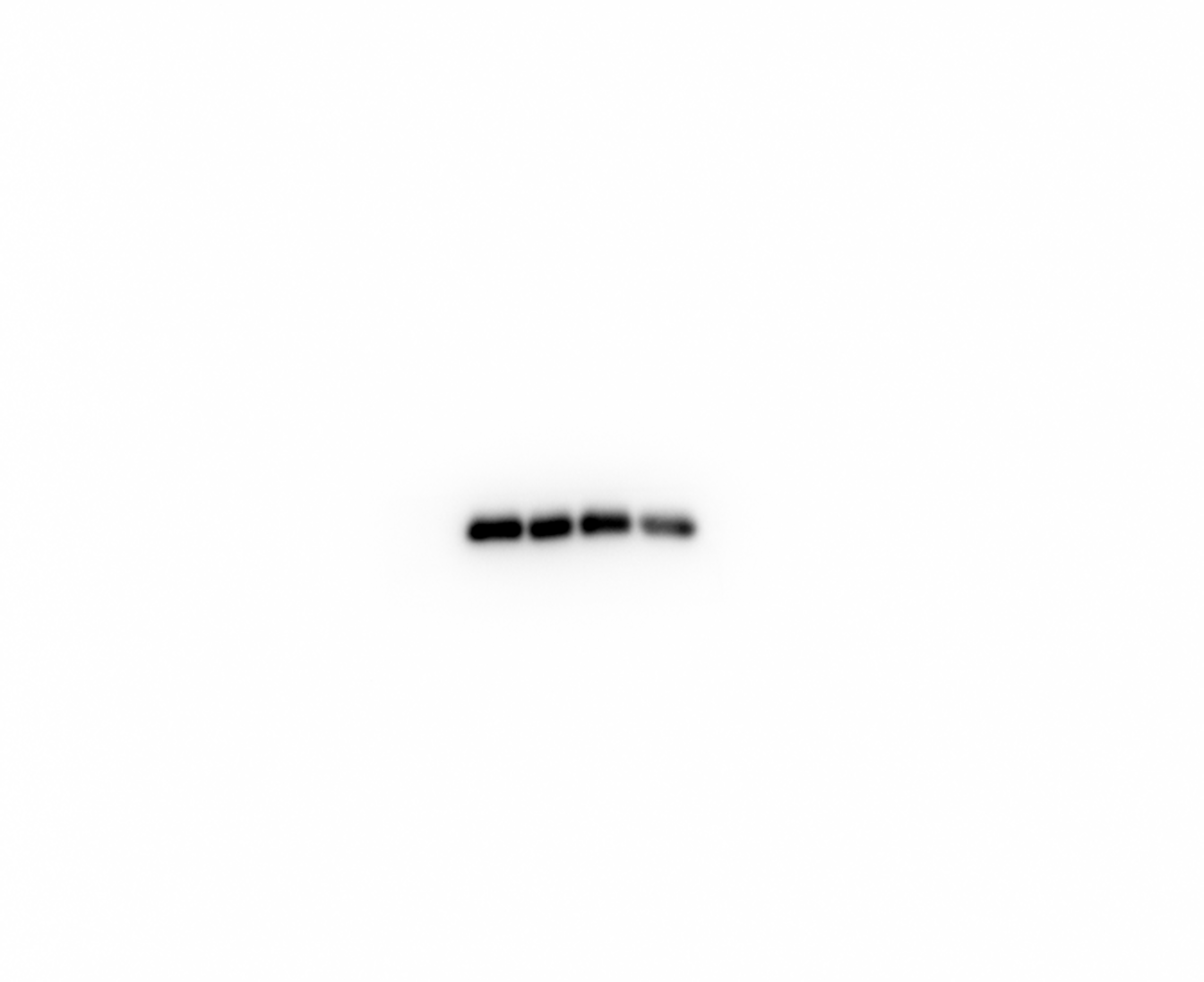


NC-C OE-C NC-LPS OE-LPS

NC-C OE-C NC-LPS OE-LPS


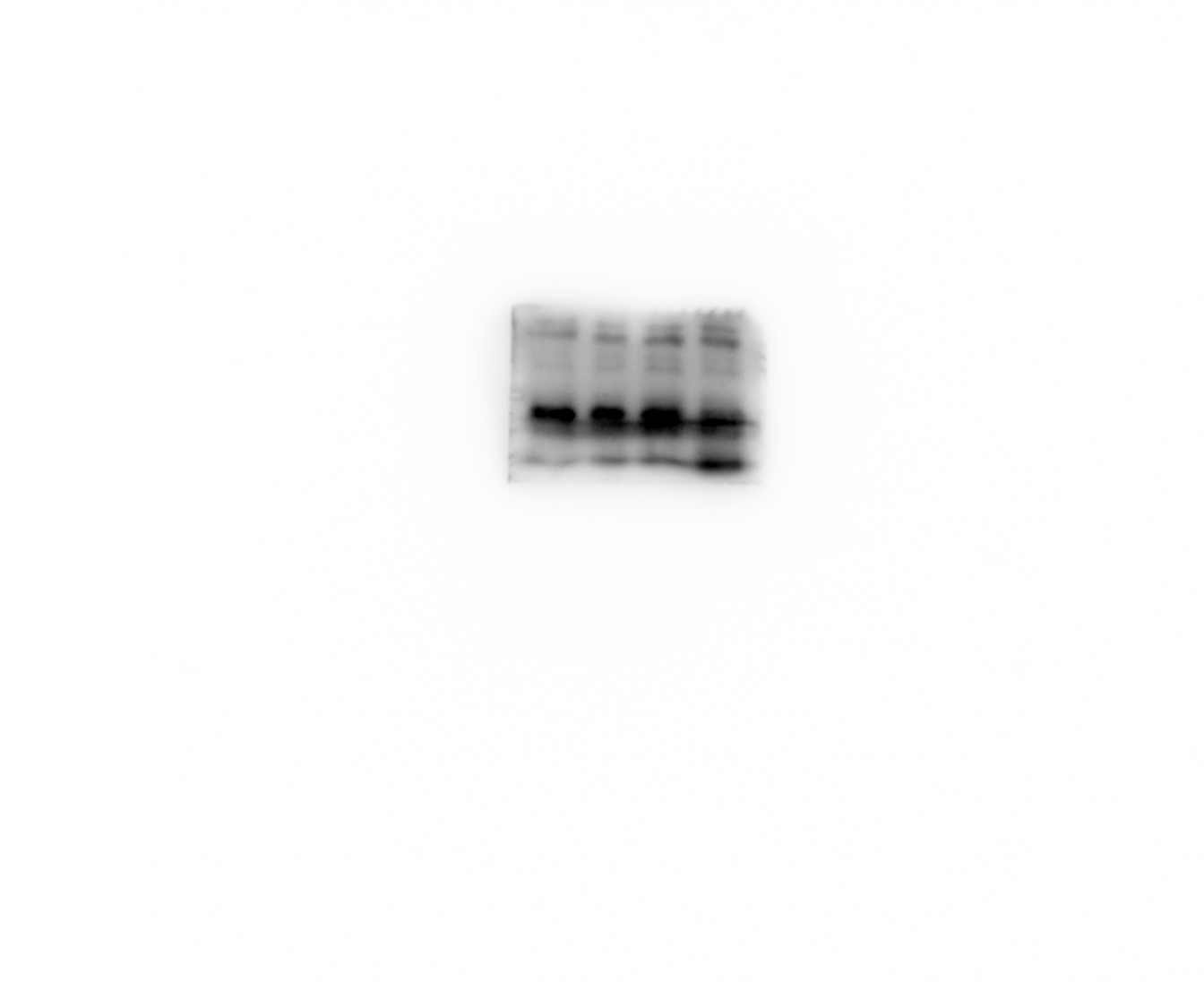


**Bcl2**

NC-C OE-C NC-LPS OE-LPS


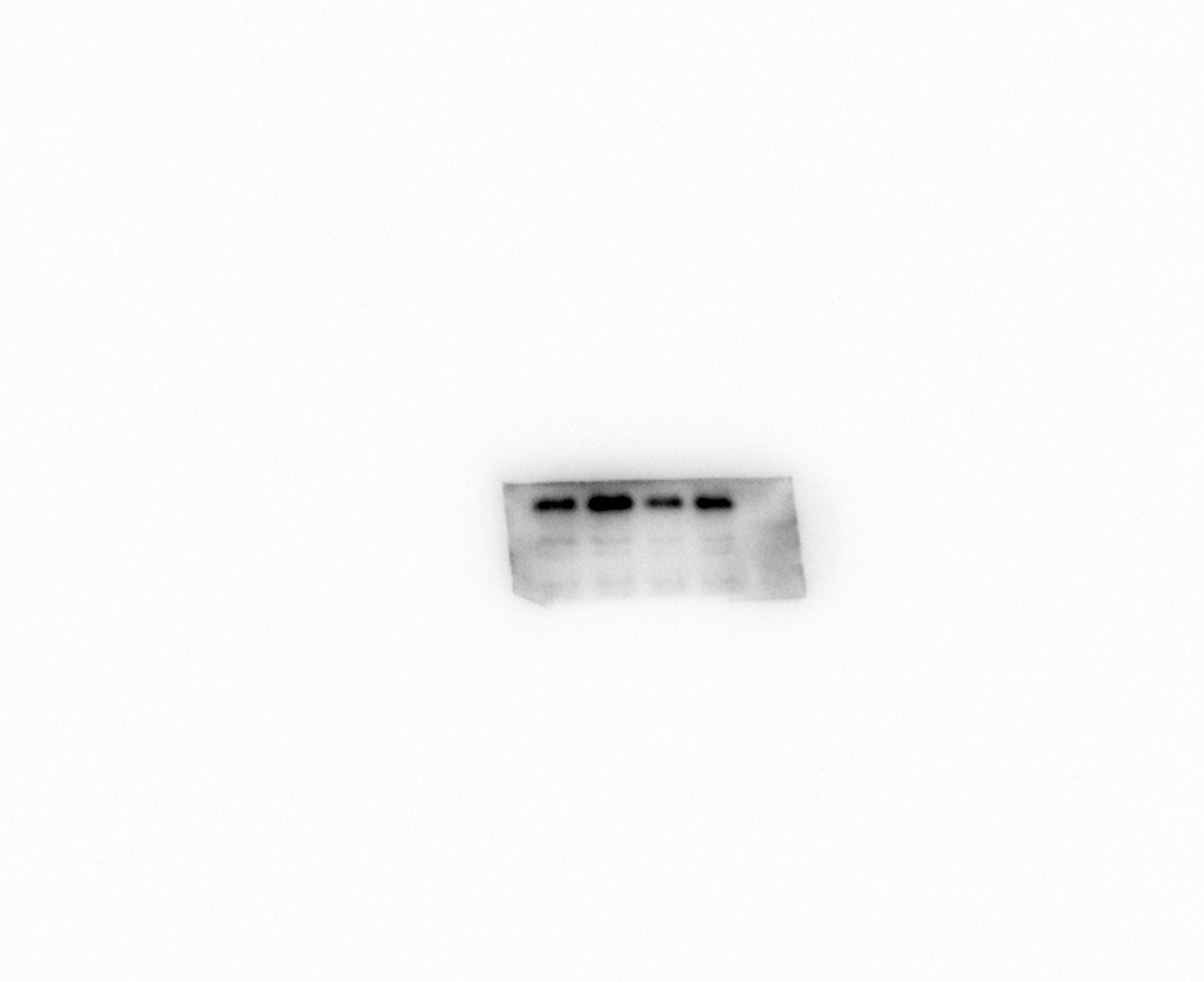


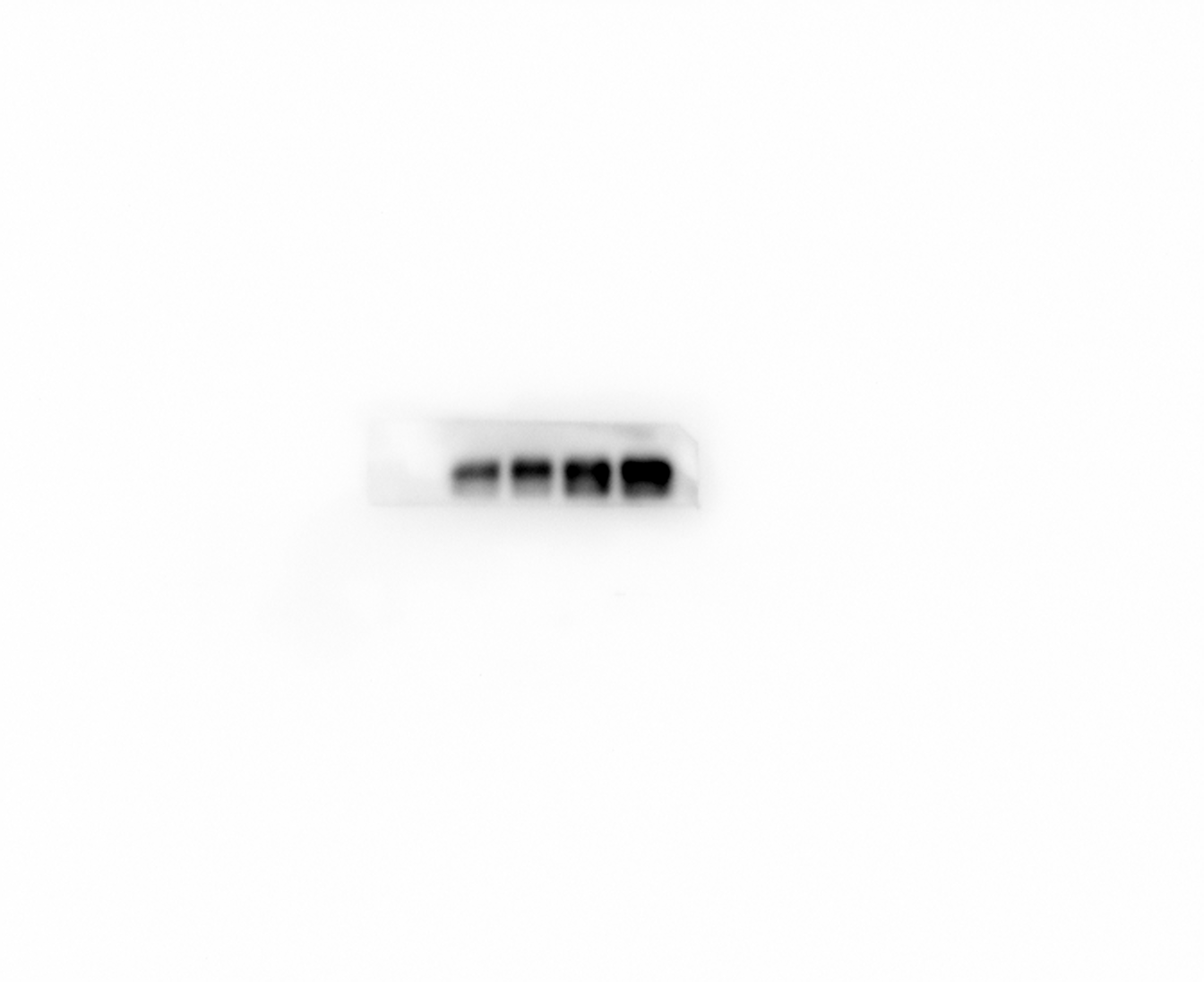


NC-C OE-C NC-LPS OE-LPS

NC-C OE-C NC-LPS OE-LPS


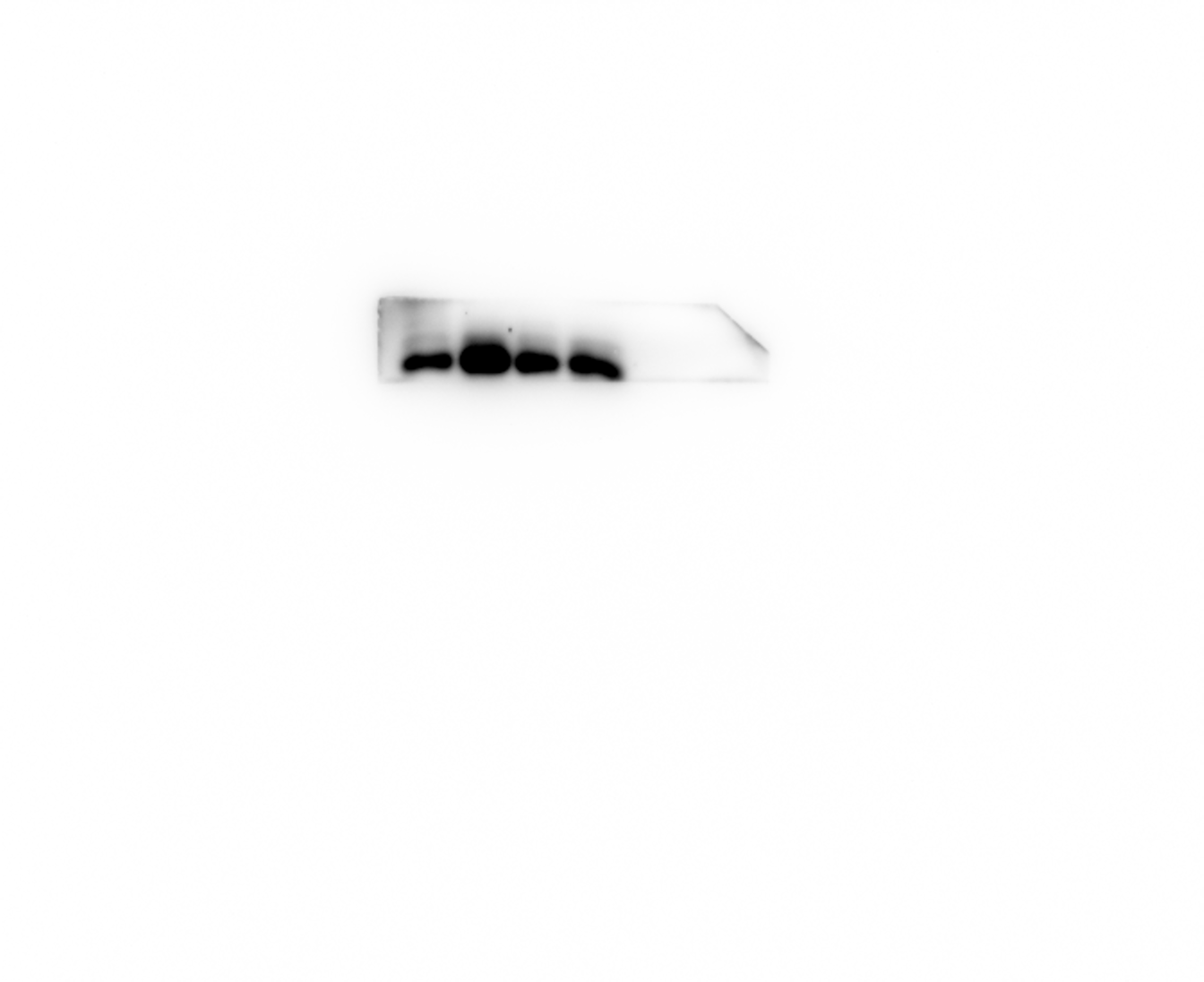


**IL-1β**

NC-C OE-C NC-LPS OE-LPS


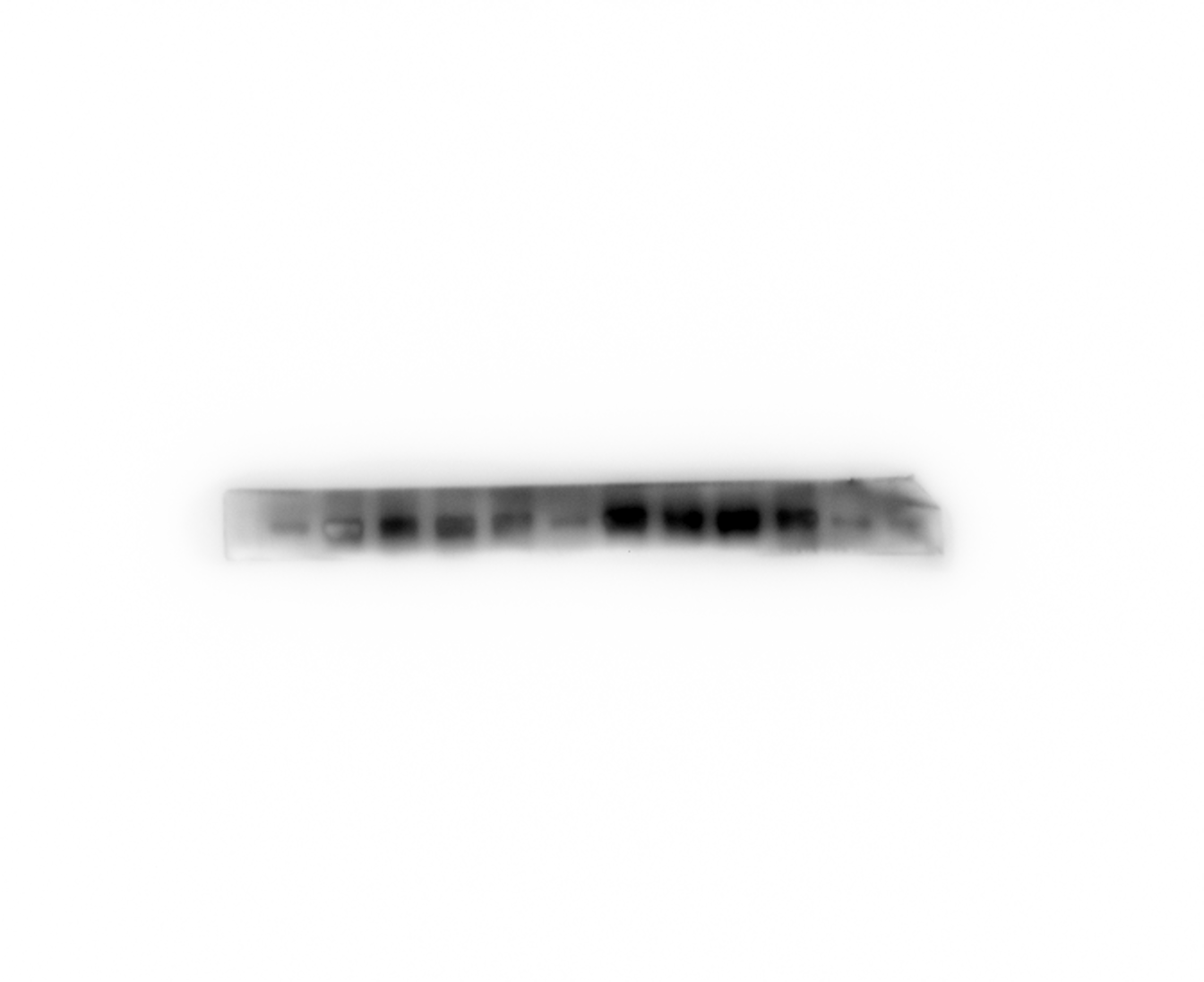


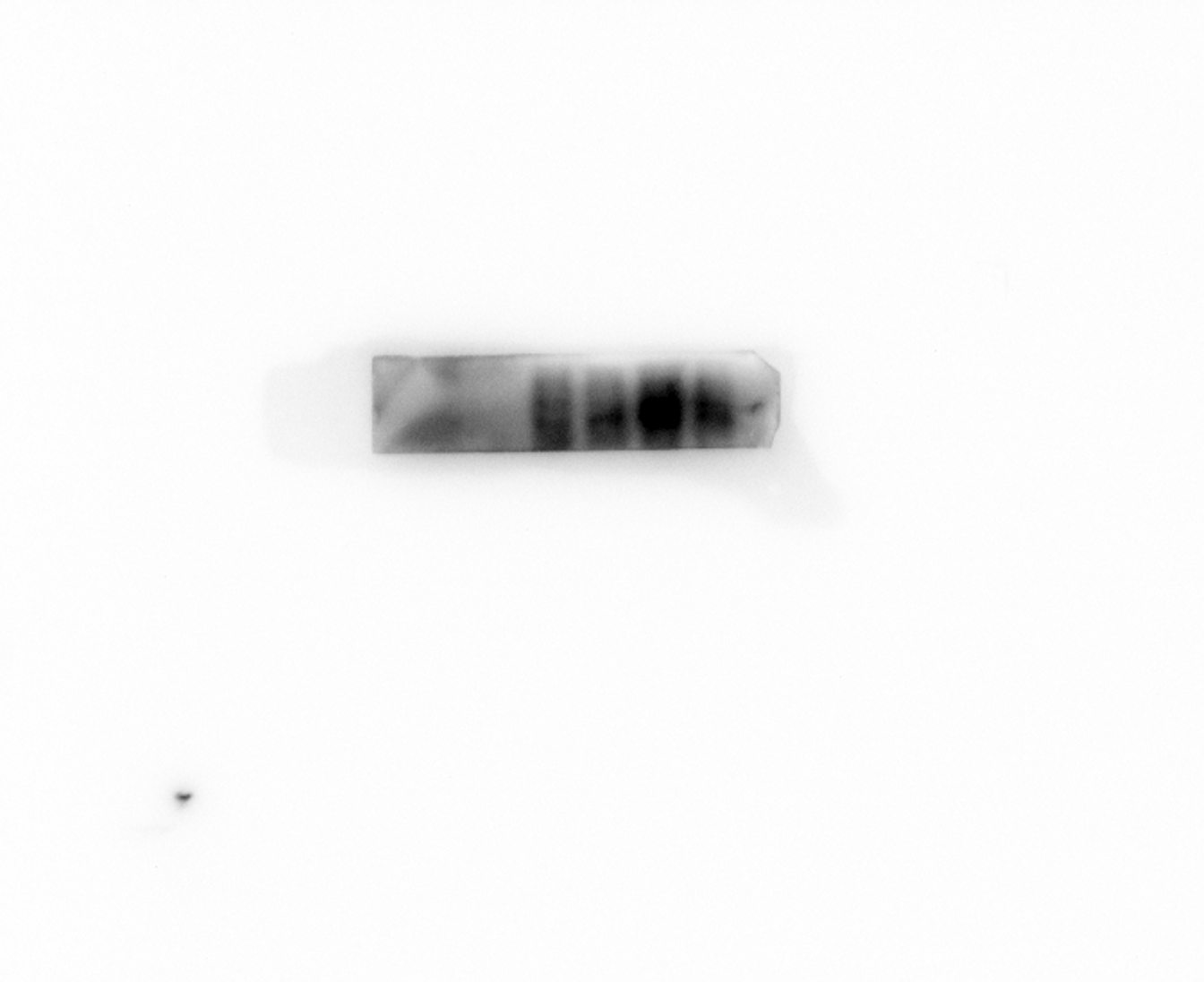


NC-C OE-C NC-LPS OE-LPS


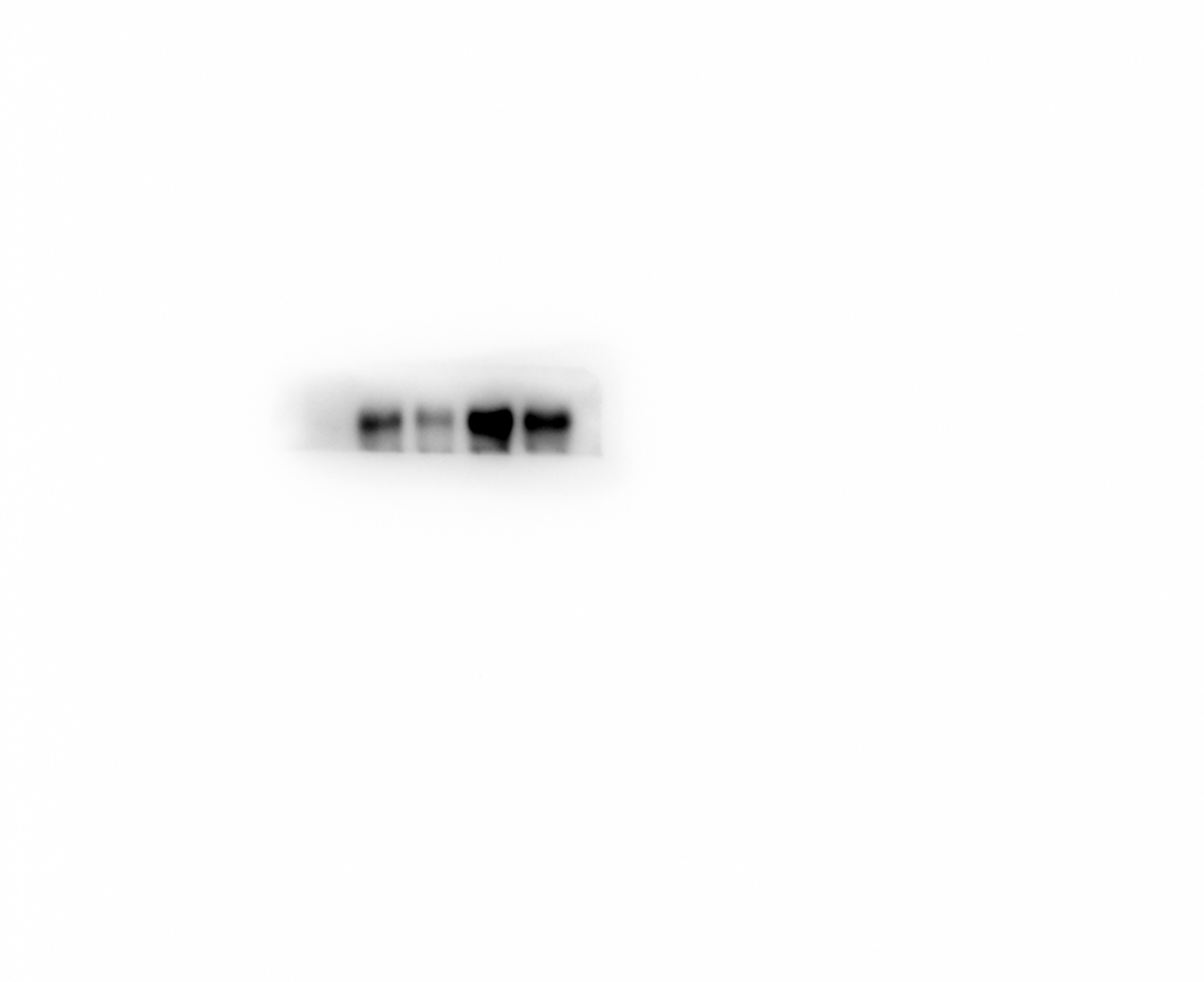


NC-C OE-C NC-LPS OE-LPS

**Cleaved-caspase3**

NC-C OE-C NC-LPS OE-LPS


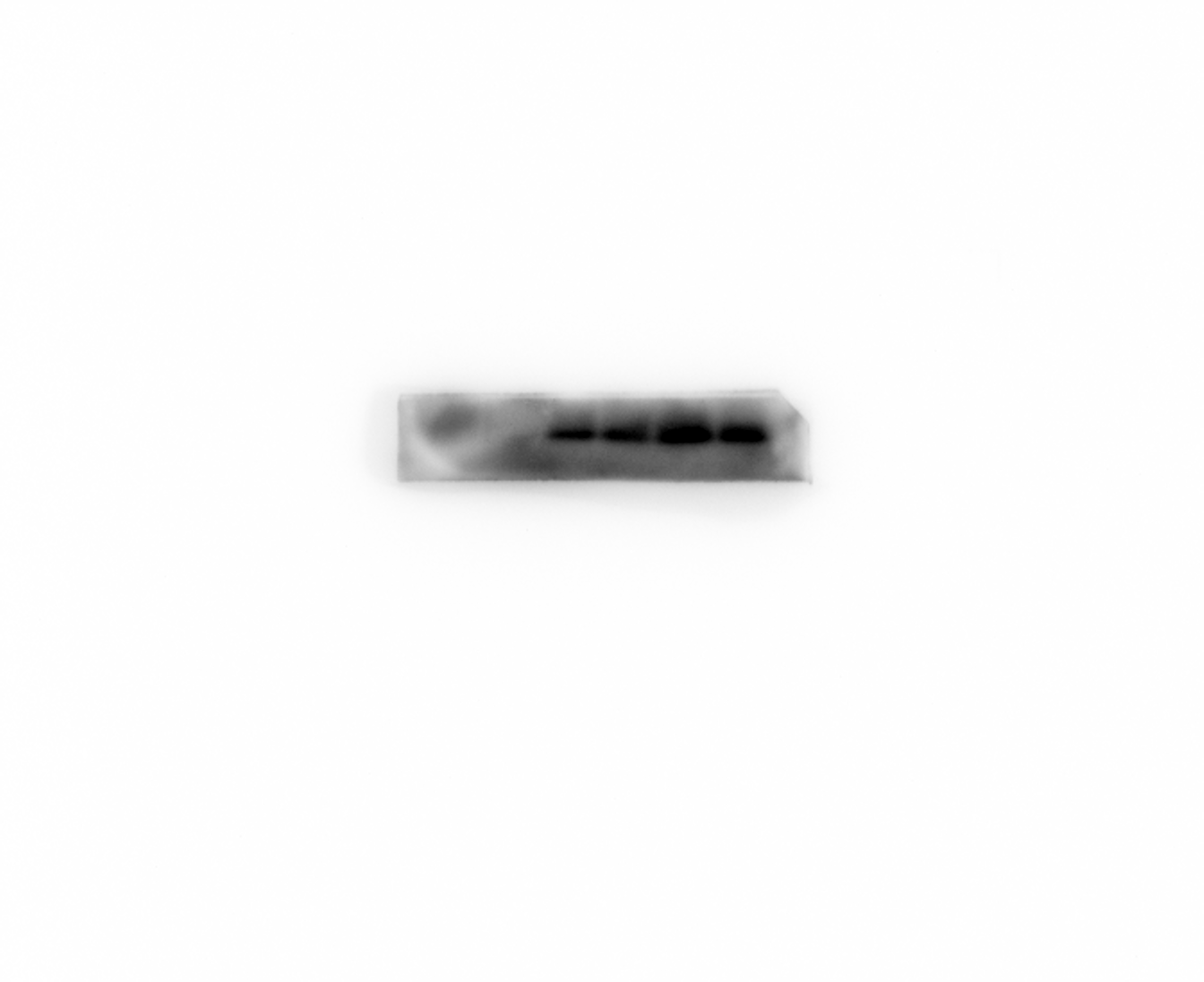


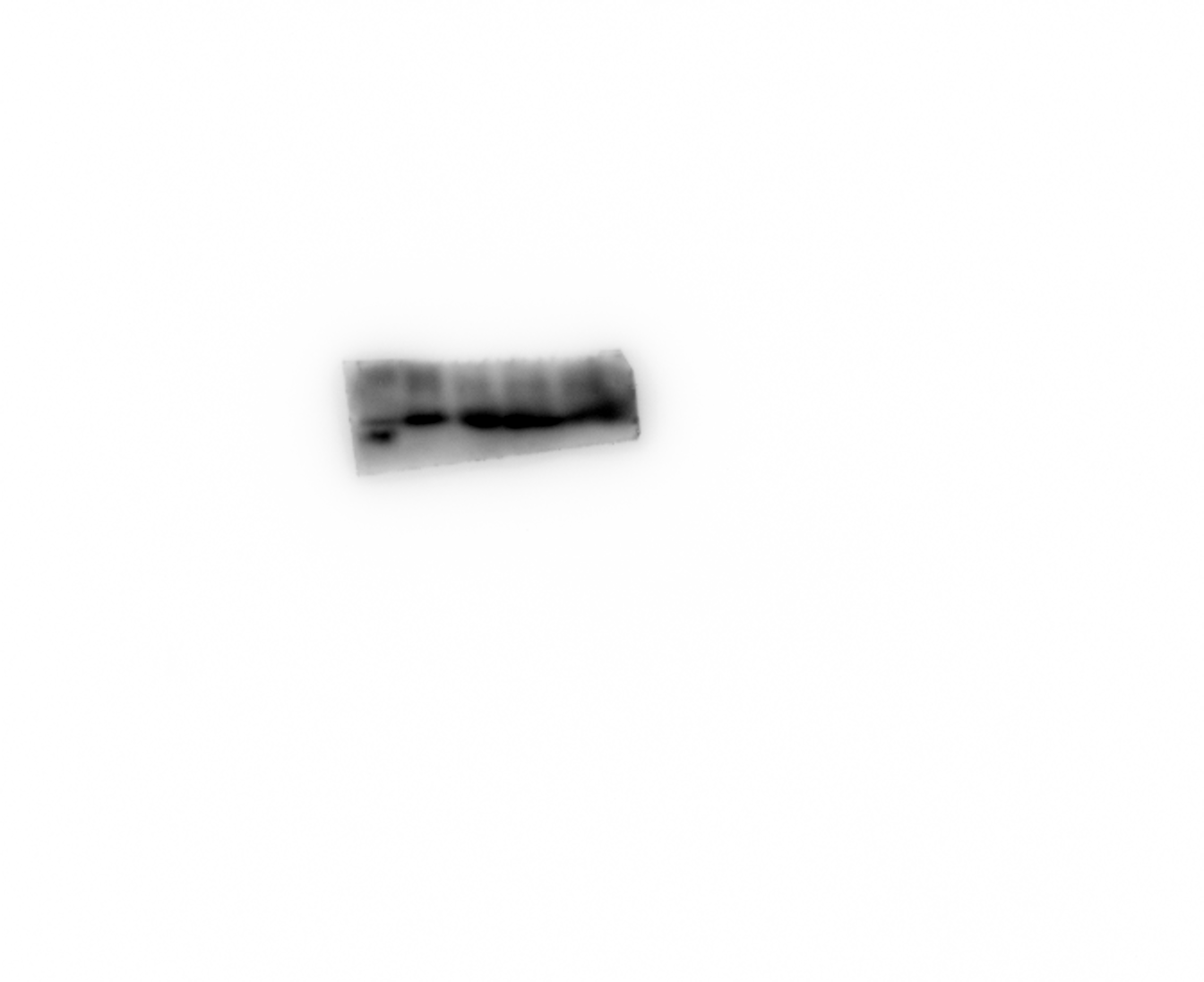


NC-C OE-C NC-LPS OE-LPS

NC-C OE-C NC-LPS OE-LPS


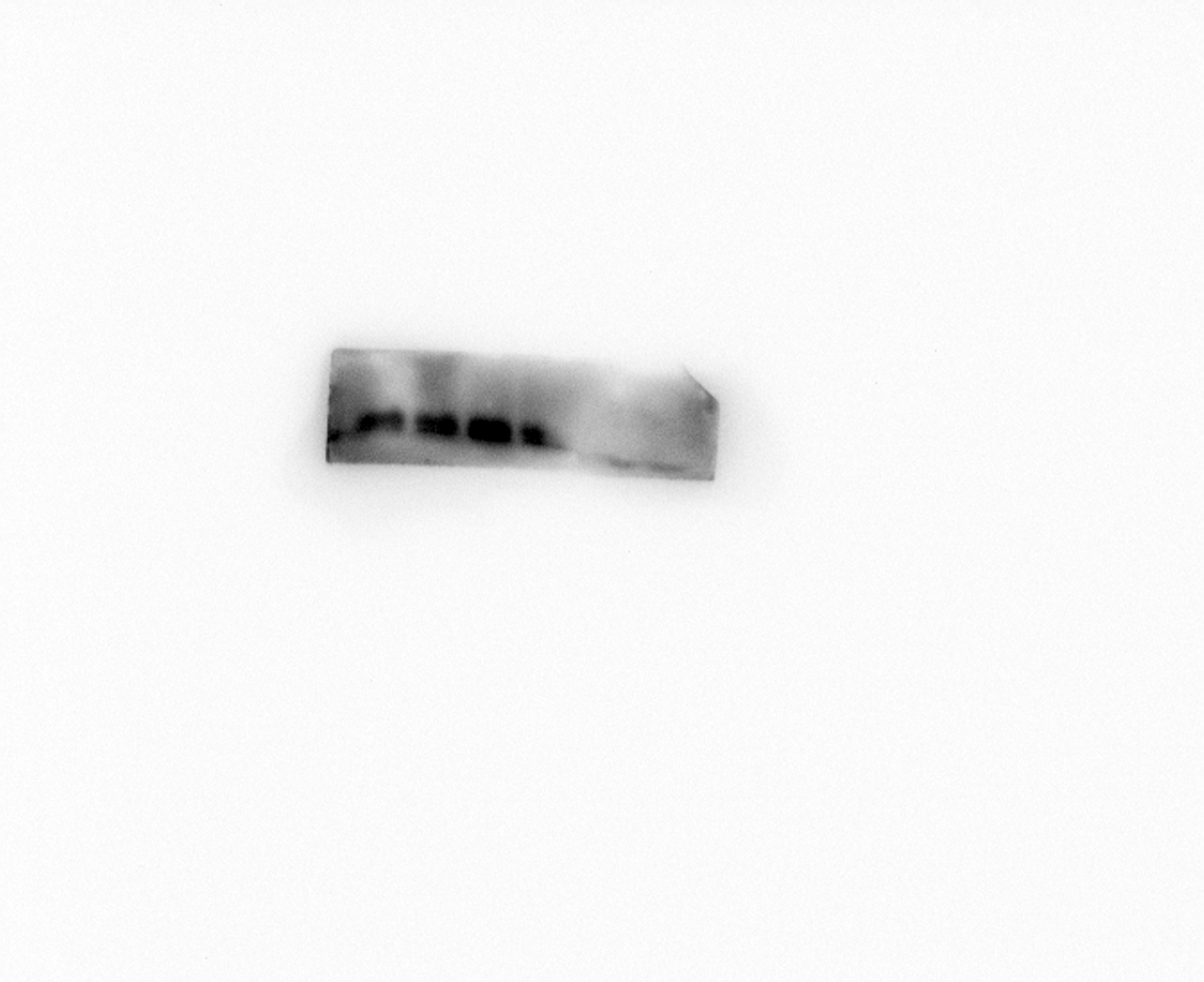

Supplement: Supplementary file 1 — Additional file 1: Supplementary materials. [file 11658_2023_488_MOESM1_ESM.docx]
